# Prescription of Lipid-Lowering and Antihypertensive Drugs Following Pictorial Information About Subclinical Atherosclerosis: A Secondary Outcome of a Randomized Clinical Trial

**DOI:** 10.1001/jamanetworkopen.2021.21683

**Published:** 2021-08-19

**Authors:** Maria Sjölander, Bo Carlberg, Margareta Norberg, Ulf Näslund, Nawi Ng

**Affiliations:** 1 Department of Public Health and Clinical Medicine, Umeå University, Umeå, Sweden; 2 Department of Integrative Medical Biology, Umeå University, Umeå, Sweden; 3 Department of Epidemiology and Global Health, Umeå University, Umeå, Sweden; 4 School of Public Health and Community Medicine, Institute of Medicine, Sahlgrenska Academy, University of Gothenburg, Göteborg, Sweden

## Abstract

**Importance:**

Preventive drugs are often underused. Carotid intima-media thickness and carotid plaques are associated with cardiovascular disease (CVD), and their detection could possibly improve estimation of the likelihood of CVD and prescription of preventive drugs.

**Objective:**

To evaluate whether pictorial information on participants’ asymptomatic atherosclerosis based on carotid ultrasonographic examinations to participants and their physicians had an effect on prescribing of lipid-lowering or antihypertensive drugs during the following 465 days.

**Design, Setting, and Participants:**

Visualization of Asymptomatic Atherosclerotic Disease for Optimum Cardiovascular Prevention is a pragmatic randomized clinical trial nested within the Västerbotten Intervention Program, a CVD screening and prevention program in Sweden with 60% to 70% participation rates and small social selection bias. A total of 4177 individuals aged 40, 50, or 60 years participating in the Västerbotten Intervention Program who had low to moderate risk of CVD were invited to enroll in this trial from April 29, 2013, to June 7, 2016. Prescriptions for all participants were monitored for 465 days after the intervention. Data analysis was conducted from December 6, 2019, to April 2, 2020.

**Interventions:**

Participants and their family physicians were randomly assigned 1:1 to receive or not receive pictorial information from carotid ultrasonographic determination of vascular age, assessed as carotid intima-media thickness and the presence of carotid plaques, combined with a follow-up call to participants by nurses.

**Main Outcomes and Measures:**

Two outcome measures of prescriptions of antihypertensive and lipid-lowering drugs within 465 days after ultrasonography was performed. Data obtained through intention-to-treat analysis are presented as proportions of individuals with a prescription among those who had no baseline prescription for agents from these drug classes.

**Results:**

Of the 4177 individuals invited to enroll, 3532 participants were randomized and included in the analysis; 1870 (52.9%) were women, 2278 (64.5%) were aged 60 years, 978 (27.7%) were 50 years, and 276 (7.8%) were 40 years. First prescriptions of lipid-lowering drugs were higher in the intervention group vs the control group among men (118 of 639 [18.5%] vs 38 of 692 [5.5%]; *P* < .001) and women (126 of 804 [15.5%] vs 38 of 817 [4.7%]; *P* < .001). There were no significant differences in the proportion with prescription of antihypertensive drugs in the intervention vs control groups after ultrasonography among men (58 of 482 [12.0%] vs 56 of 528 [10.6%]; *P* = .47) and women (60 of 612 [9.8%] vs 64 of 615 [10.4%]; *P* = .73).

**Conclusions and Relevance:**

The findings of this trial demonstrate that provision of pictorial information on vascular age and carotid plaques based on the results of ultrasonographic examination increased physician prescription of lipid-lowering drugs but not antihypertensive drugs within the following 465 days.

**Trial Registration:**

ClinicalTrials.gov Identifier: NCT01849575

## Key Points

**Question:**

Is provision of pictorial information on asymptomatic atherosclerosis to physicians and patients associated with prescribing of lipid-lowering or antihypertensive drugs during the following 465 days?

**Findings:**

In this randomized clinical trial including 3532 participants with low to moderate cardiovascular disease risk, use of pictorial information on carotid intima-media thickness and carotid plaques based on ultrasonographic examination was evaluated. Physician prescription increased for lipid-lowering medications but not for antihypertensive drugs.

**Meaning:**

The findings of this randomized clinical trial demonstrate that information on carotid intima-media thickness and the presence of carotid plaques can improve prescription of lipid-lowering drugs.

## Teaser

This randomized clinical trial examines the effect of the use of pictorial information including carotid ultrasonographic images with explanation on prescribing of lipid-lowering and antihypertensive drugs.

## Introduction

Both therapeutic drugs and lifestyle changes are effective in prevention of cardiovascular disease (CVD), although preventive drugs are often underused.^1^ Increased risk is often identified and communicated using traditional screening tools (eg, European Systematic Coronary Risk Evaluation)^2^ and Framingham risk score).^3^ Although these measures are useful in identifying individuals with high risk, they are less sensitive in asymptomatic individuals with low to moderate risk among whom most cardiovascular events occur.

Both increased carotid intima-media thickness (cIMT) and presence of carotid plaques are associated with CVD.^4,5^ Ultrasonographic examinations for detecting carotid plaques and increased cIMT can therefore be used to improve the estimation of the likelihood of future CVD.^6^ The association between visual representations of asymptomatic diseases and the initiation of drug treatment has been evaluated in other medical fields.^7,8^ However, the evidence for use of pictorial information from cardiovascular imaging combined with conventional risk factor information to improve CVD risk assessment and prescription of preventive medications is limited.^9^ Randomized clinical trials on the use of pictorial information from cardiovascular imaging are few and often small, or imaging results are given verbally or as text or numbers.^10,11^

In the Visualization of Asymptomatic Atherosclerotic Disease for Optimum Cardiovascular Prevention (VIPVIZA), a randomized clinical trial nested in the Västerbotten Intervention Program in Sweden (VIP), persons with low to moderate CVD risk and their physicians were randomized to receive pictorial information of participants’ carotid ultrasonographic results combined with written information about atherosclerosis, and participants also received a follow-up phone call from a nurse. The results of the VIPVIZA primary outcome at 1 year follow-up showed significant differences in the Framingham risk score and European Systematic Coronary Risk Evaluation between the intervention and control participants.^12^ We hypothesized that the intervention would influence physicians’ prescription of lipid-lowering and antihypertensive drugs.

In this secondary analysis of VIPVIZA, we aimed to assess whether the proportion of participants with a first prescription of lipid-lowering or antihypertensive drugs differed between intervention and control groups during 465 days following the carotid ultrasonographic examination and whether first prescriptions were related to the information about carotid plaques. Furthermore, we investigated whether factors other than vascular age and plaques were associated with prescription of lipid-lowering or antihypertensive drugs.

## Methods

VIPVIZA has been approved by the Regional Ethical Review Board, Umeå University. All participants have given informed written consent; no financial compensation was provided. The trial protocol and statistical analysis plan are available in Supplement 1. This study followed the Consolidated Standards of Reporting Trials (CONSORT) reporting guideline for randomized clinical trials.

The pragmatic randomized controlled study VIPVIZA was set up within the VIP. The VIP is a population-based program for CVD prevention in Västerbotten County, Sweden. The VIP has been running for over 25 years and the participation rates during 2013-2016 were 60%-70% with only small social selection bias.^13^ The VIP invites all individuals in the county the year they turn 40, 50 and 60 years old to risk factor screening and individual health promotion at their primary health care center. Follow-up and pharmacologic treatment are provided according to clinical guidelines. From April 29, 2013, to June 7, 2016, participants who went through the VIP program were thereafter invited to enroll in VIPVIZA if they fulfilled the inclusion criteria. Data analysis was conducted from December 6, 2019, to April 2, 2020.

Inclusion criteria for VIPVIZA were (1) age 40 years and first-degree relative with a history of CVD at age less than 60 years, (2) age 50 years and at least 1 designated risk factor (smoking, diabetes, hypertension, serum low-density lipoprotein cholesterol level ≥174 mg/dL [to convert to millimoles per liter, multiply by 0.0259], abdominal obesity, or first-degree relative with a history of CVD at age <60 years), or (3) age 60 years.

All individuals who agreed to participate in VIPVIZA underwent a carotid ultrasonographic examination but did not receive any information on the results during the examination. Examinations were performed by trained sonographers from the Heart Centre, Umeå University Hospital, according to a standardized protocol (additional information on the ultrasonographic examinations is available in the eAppendix in Supplement 2).^12^ A list with a computer-generated random allocation sequence (equal probability) was prepared in advance, and participants were allocated consecutively before examination. The allocation was concealed from both participants and examiners. Participants who were found to have stenosis greater than or equal to 50% of the carotid lumen (n = 22) were excluded from the study and referred to specialized care for evaluation and treatment.

### The VIPVIZA Intervention

The intervention consisted of information to the participants and their primary care physicians about the ultrasonography results sent by mail within 2 weeks after the examination. The information was pictorial and included a simplified picture of the scan (eFigure 1 in Supplement 2), a red dot to indicate plaque or a green dot to indicate no plaque for each side. A gauge ranging from green through yellow and orange to red indicated vascular age, with green presented as being at least 10 years younger than the chronological age and red as at least 10 years older. Vascular age was based on the participant’s cIMT in relation to the cIMT of persons the same age and sex in the reference population in the Atherosclerosis Risk in Communities study.^14^

The information also included a brief description of atherosclerosis as a dynamic process and possibilities for prevention. Within 2 to 4 weeks, a specially trained VIPVIZA nurse made a telephone call to each participant in the intervention group to ascertain that they understood the results and provide any needed information. No phone calls were made to primary care physicians. The nurse worked at the VIPVIZA study center and did not interact with the primary health care center. Intervention group participants’ primary care physicians received identical ultrasonography results by mail as well as information that the presence of plaques indicates a very high risk of CVD.^5^ The identical ultrasonography report was re-sent to the participants 6 months after the examination.

No information about the ultrasonography results were given to control group participants and their physicians, and these results were also not available within the health care information system. Participants in both study groups obtained CVD prevention measures within the usual primary care practices. VIPVIZA also aimed to evaluate the effect of the intervention to improve adherence to prevention guidelines among both participants and physicians. More details of the VIPVIZA have been described elsewhere.^12^

The data set linked information from examinations and questionnaires in VIP and VIPVIZA at baseline. The VIP data included blood lipid levels, blood pressure, and questions on health, socioeconomic status, and lifestyle. VIPVIZA baseline data included cIMT and the presence of plaques. Data on prescribed drugs were retrieved from the computerized medical records in the Region Västerbotten database.

Participants were considered to be prescribed a lipid-lowering drug (Anatomical Therapeutic Classification code C10) or antihypertensive drug (Anatomical Therapeutic Classification codes C03A, C07, C08, and C09) at baseline if they had received at least 1 prescription within 465 days before the date of the ultrasonographic examination or at follow-up if they had received at least 1 prescription within 465 days after the ultrasonographic examination. Data from the follow-up are presented as the proportion of individuals with a first prescription among those without a baseline prescription.

A period of 465 days was chosen because prescriptions in Sweden often cover 400 days’ supply. Operational definitions of variables are reported in eTable 1 in Supplement 2. The CONSORT flow diagram for VIPVIZA has been published,^12^ and the diagram with further information specific to the present trial is shown in eFigure 2 in Supplement 2.

### Statistical Analysis

A sample size of 1500 individuals per group would allow identification of minimum detectable change of 0.02 mm in cIMT from baseline to 3-year follow-up with a power of 80% and precision of 5%. Estimation of the effects of our primary and secondary outcomes would maintain the same level of power and precision in our study with even smaller sample sizes.^12^ *P* values <.05 were used to signify statistical significance on 2-sided hypotheses testing. Baseline data were analyzed separately for men and women. Using intention-to-treat analysis, we compared the proportion of participants with prescriptions in the intervention and control groups using the χ^2^ test. We performed subgroup analysis based on the presence of plaques. Multivariable logistic regression models were used to compare the odds of prescriptions among participants in the intervention compared with the control group and to investigate which factors, other than plaque and cIMT, were associated with prescription. Independent variables in the logistic regression models included age, sex, educational level, diabetes, blood pressure greater than or equal to 140/90 mm Hg, serum cholesterol levels, prescription of antihypertensive or lipid-lowering drugs at baseline, and year of baseline data collection. Information about plaque and cIMT were included in separate analyses for the intervention group only, because this information was concealed from participants and physicians in the control group. Data were analyzed using SPSS Statistics, version 26 (IBM Corp).

## Results

From April 29, 2013, to June 7, 2016, 3532 participants were recruited to enroll in VIPVIZA, of whom 1870 (52.9%) were women, 2278 (64.5%) were aged 60 years, 978 (27.7%) were 50 years, and 276 (7.8%) were 40 years. Higher levels of baseline total cholesterol levels were observed in women (men, 213 mg/dL vs women, 220 mg/dL; *P* < .001), but only 12.8% of women had been prescribed a lipid-lowering drug compared with 19.9% of men (*P* < .001). A larger proportion of men had blood pressure levels greater than or equal to 140/90 mm Hg (42.2% vs 28.2% for women; *P* < .001) and had a prescription for an antihypertensive drug at baseline (39.2% vs 34.4%; *P* = .003). Other baseline characteristics are presented in Table 1. The level of missing data was generally low, with the highest proportion (1.5%) being for diabetes.

**Table 1.** Baseline Characteristics of the 3532 Participants

Characteristic^a^	No. (%)			
	Men		Women	
	Control (n = 853)	Intervention (n = 809)	Control (n = 930)	Intervention (n = 940)
Age, y				
40	73 (8.6)	63 (7.8)	69 (7.4)	71 (7.6)
50	250 (29.3)	222 (27.4)	242 (26.0)	264 (28.1)
60	530 (62.1)	524 (64.8)	619 (66.6)	605 (64.4)
High educational level	240 (28.3)	222 (27.6)	385 (42.1)	370 (39.7)
Previous myocardial infarction	24 (2.8)	36 (4.5)	6 (0.6)	11 (1.2)
Diabetes	63 (7.4)	75 (9.4)	60 (6.6)	46 (5.0)
Blood pressure, mean (SD), mm Hg				
Systolic	131.9 (15.3)	132.4 (16.5)	126.8 (16.1)	127.0 (16.4)
Diastolic	84.7 (10.4)	85.1 (10.9)	80.7 (9.8)	80.6 (10.2)
Hypertension^b^	488 (57.2)	490 (60.6)	441 (47.4)	455 (48.4)
Prescription of antihypertensive drug at baseline	325 (38.1)	327 (40.4)	315 (33.9)	328 (34.9)
Total cholesterol, mg/dL^c^				
<193	261 (30.6)	258 (31.9)	214 (23.0)	231 (24.6)
193-250	432 (50.6)	399 (49.3)	517 (55.6)	501 (53.3)
>250	160 (18.8)	152 (18.8)	199 (21.4)	208 (22.1)
LDL-C ≥97 mg/dL	696 (81.6)	668 (82.6)	813 (87.4)	821 (87.3)
Prescription of lipid-lowering drug at baseline	161 (18.9)	170 (21.0)	113 (12.2)	126 (13.4)
SCORE risk estimates, median (range)	1.72 (0.12-14.89)	1.76 (0.14-13.02)	0.64 (0.04-3.98)	0.61 (0.04-4.22)
Year when participants were recruited to VIPVIZA^d^				
First year	282 (33.1)	240 (29.7)	257 (27.6)	308 (32.8)
Second year	259 (30.4)	217 (26.8)	278 (29.9)	292 (31.1)
Third year	312 (36.6)	352 (43.5)	395 (42.5)	340 (36.2)
Vascular age^e^				
Green/yellow	246 (28.8)	237 (29.3)	245 (26.3)	235 (25.0)
Orange/red	607 (71.2)	572 (70.7)	685 (73.7)	705 (75.0)
Carotid plaque	439 (51.5)	411 (50.8)	373 (40.1)	356 (38.0)

Abbreviations: LDL-C, low-density lipoprotein cholesterol; SCORE, European Systematic Coronary Risk Evaluation; VIPVIZA, Visualization of Asymptomatic Atherosclerotic Disease for Optimum Cardiovascular Prevention.

SI conversion: To convert LDL-C and total cholesterol to millimoles per liter, multiply by 0.0259.

^a^ Missing in the data set were 35 individuals (1%) on educational level, 30 (0.8%) on previous myocardial infarction, 54 (1.5%) on diabetes, and 2 (0.1%) on carotid plaque.

^b^ Systolic blood pressure greater than or equal to 140 mm Hg and/or diastolic blood pressure greater than or equal to 90 mm Hg or antihypertensive drug treatment at baseline.

^c^ Including individuals receiving lipid-lowering treatment.

^d^ First year: April 2013-June 2014, second year: July 2014-June 2015, and third year: July 2015-June 2016.

^e^ Vascular age was indicated by a gauge from green (carotid intima-media thickness in persons at least 10 years younger than their chronologic age) through yellow and orange to red (carotid intima-media thickness in persons at least 10 years older than their chronologic age). Information about plaques and vascular age were not disclosed to the control group.

Prescription of lipid-lowering drugs after ultrasonography to participants without a baseline prescription for lipid-lowering drugs was more common in the intervention group among both men and women: 18.5% (118 of 639) of men in the intervention group compared with 5.5% (38 of 692) in the control group (*P* < .001), and 15.5% (126 of 814) of women in the intervention group compared with 4.7% (38 of 817) in the control group (*P* < .001) were prescribed lipid-lowering drugs. Only 2 of all prescriptions for a lipid-lowering drug were not a statin. There were no significant differences in the proportion of participants with a first prescription for antihypertensive drugs between the intervention and control group among either men or women: 12.0% (58 of 482) of men in the intervention group compared with 10.6% (56 of 528) in the control group (*P* = .47), and 9.8% (60 of 612) of women in the intervention group compared with 10.4% (64 of 615) in the control group (*P* = .73) were prescribed antihypertensive drugs. In the logistic regression models, we observed an association between the intervention and prescription of lipid-lowering drugs but not with antihypertensive drugs (Table 2).

**Table 2.** Logistic Regression Comparing Participants With Prescription After Ultrasonography vs Participants Without Prescription

Variable	Lipid-lowering drugs			Antihypertensive drugs		
	No. (%)		Multiple logistic regression (n = 2888), OR (95% CI)	No. (%)		Multiple logistic regression (n = 2178), OR (95% CI)
	No prescription before or after ultrasonography (n = 2642)^a^	No prescription before but prescription after ultrasonography (n = 320)		No prescription before or after ultrasonography (n = 1999)^a^	No prescription before but prescription after ultrasonography (n = 238)	
Group						
Control	1433 (95.0)	76 (5.0)	1 [Reference]	1023 (89.5)	120 (10.5)	1 [Reference]
Intervention	1209 (83.2)	244 (16.8)	4.04 (3.07-5.33)	976 (89.2)	118 (10.8)	0.97 (0.73-1.30)
Age, y						
40	249 (94.0)	16 (6.0)	1 [Reference]	221 (94.0)	14 (6.0)	1 [Reference]
50	789 (89.6)	92 (10.4)	1.10 (0.62-1.97)	571 (88.3)	76 (11.7)	1.98 (1.05-3.75)
60	1604 (88.3)	212 (11.7)	1.17 (0.67-2.04)	1207 (89.1)	148 (10.9)	1.78 (0.96-3.30)
Sex						
Men	1175 (88.3)	156 (11.7)	1 [Reference]	896 (88.7)	114 (11.3)	1 [Reference]
Women	1467 (89.9)	164 (10.1)	0.82 (0.64-1.05)	1103 (89.9)	124 (10.1)	1.20 (0.89-1.61)
Educational level						
Low/middle	1663 (89.0)	206 (11.0)	1 [Reference]	1213 (88.4)	159 (11.6)	1 [Reference]
High	949 (89.3)	114 (10.7)	1.19 (0.91-1.54)	763 (90.7)	78 (9.3)	1.01 (0.74-1.37)
Diabetes						
No	2503 (89.5)	295 (10.5)	1 [Reference]	1897 (89.7)	218 (10.3)	1 [Reference]
Yes	95 (81.2)	22 (18.8%)	2.52 (1.50-4.23)	66 (78.6)	18 (21.4)	2.15 (1.17-3.93)
Blood pressure ≥140/90 mm Hg						
No	1780 (90.5)	186 (9.5)	1 [Reference]	1573 (94.9)	85 (5.1)	1 [Reference]
Yes	862 (86.5)	134 (13.5)	1.12 (0.86-1.47)	426 (73.6)	153 (26.4)	6.73 (5.00-9.05)
Prescription for antihypertensive drug at baseline						
No	1885 (90.7)	193 (9.3)	1 [Reference]	NA	NA	NA
Yes	757 (85.6)	127 (14.4)	1.70 (1.30-2.23)	NA	NA	NA
Prescription for lipid-lowering drug at baseline						
No	NA	NA	NA	1864 (89.7)	214 (10.3)	1 [Reference]
Yes	NA	NA	NA	135 (84.9)	24 (15.1)	1.50 (0.90-2.50)
Serum cholesterol, mg/dL						
<193	636 (94.5)	37 (5.5)	1 [Reference]	463 (90.3)	50 (9.7)	1 [Reference]
193-250	1541 (90.8)	157 (9.2)	1.90 (1.29-2.81)	1079 (88.7)	137 (11.3)	1.07 (0.74-1.56)
>250	465 (78.7)	126 (21.3)	5.87 (3.86-8.94)	457 (90.0)	51 (10.0)	0.83 (0.53-1.30)
Year of the study^b^						
First year	845 (92.2)	71 (7.8)	1 [Reference]	621 (90.0)	69 (10.0)	1 [Reference]
Second year	768 (88.3)	102 (11.7)	1.74 (1.24-2.44)	618 (90.6)	64 (9.4)	0.87 (0.60-1.28)
Third year	1029 (87.5)	147 (12.5)	1.86 (1.36-2.55)	760 (87.9)	105 (12.1)	1.13 (.080-1.59)

Abbreviation: OR, odds ratio.

SI conversion value: To convert low-density lipoprotein and total cholesterol to millimoles per liter, multiply by 0.0259.

^a^ Reference group.

^b^ First year: April 2013-June 2014, second year: July 2014-June 2015, third year: July 2015-June 2016.

**Figure 1.**
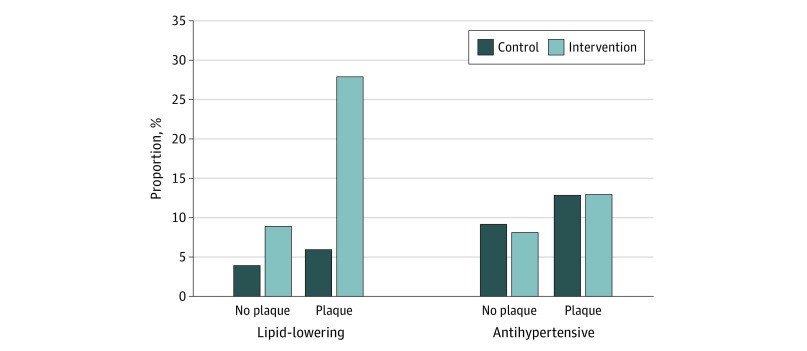
Proportion of Women With a First Prescription for a Lipid-Lowering or Antihypertensive Drug The results are presented for women with and without carotid plaques. Information from ultrasonography was not disclosed to the control group.

**Figure 2.**
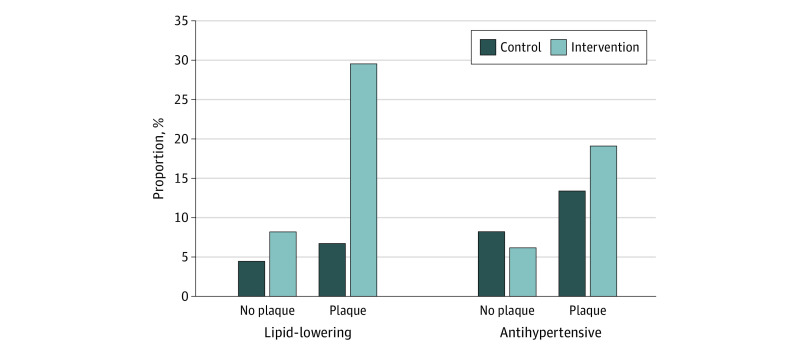
Proportion of Men With a First Prescription for a Lipid-Lowering or Antihypertensive Drug The results are presented for men with and without carotid plaques. Information from ultrasonography was not disclosed to the control group.

When stratified for the presence of plaque, the proportion with a first prescription for a lipid-lowering drug was higher in the intervention group compared with controls, both among those with plaques (for men: control, 6.7% [22 of 328] vs intervention, 29.5% [91 of 308]; *P* < .001; for women: control, 5.9% [18 of 307] vs intervention, 27.9% [79 of 283]; *P* < .001) and those without plaques (for men: control, 4.4% [16 of 364] vs intervention, 8.2% [27 of 331]; *P* = .04; for women: control, 3.9% [20 of 510] vs intervention, 8.9% [47 of 529]; *P* = .001). However, the proportion was more than 3 times higher in participants with plaques (Figure 1 and Figure 2). There were no significant differences in prescription of antihypertensive drugs when stratifying for plaque in men or women (Figure 1 and Figure 2). Results stratified for both the presence of plaque and vascular age are presented in eTable 2 and eTable 3 in Supplement 2). In the separate regression models for the intervention group, prescription of lipid-lowering drugs was associated with carotid plaques (odds ratio [OR], 3.77; 95% CI, 2.73-5.22) and vascular age (OR, 1.57; 95% CI, 1.08-2.29), but prescription of antihypertensive drugs was associated only with plaques (OR, 2.32; 95% CI, 1.48-3.64) (Table 3).

**Table 3.** Factors Associated With Prescription of Lipid-Lowering and Antihypertensive Drugs in the Intervention Group After Carotid Ultrasonography

Variable	Lipid-lowering drugs (n = 1453)			Antihypertensive drugs (n = 1094)		
	No. (%)		Multiple logistic regression (n = 1416]), OR (95% CI)	No. (%)		Multiple logistic regression (n = 1067), OR (95% CI)
	No prescription before or after ultrasonography (n = 1209)	No prescription before but prescription after ultrasonography (n = 244)		No prescription before or after ultrasonography (n = 976)	No prescription before but prescription after ultrasonography (n = 118)	
Age, y						
40	116 (90.6)	12 (9.4)	1 [Reference]	108 (92.3)	9 (7.7)	1 [Reference]
50	368 (84.4)	68 (15.6)	0.98 (0.48-2.00)	271 (88.0)	37 (12.0)	1.48 (0.62-3.50)
60	725 (81.6)	164 (18.4)	0.89 (0.44-1.77)	597 (89.2)	72 (10.8)	1.11 (0.48-2.60)
Sex						
Men	521 (81.5)	118 (18.5)	1 [Reference]	424 (88.0)	58 (12.0)	1 [Reference]
Women	688 (84.5)	126 (15.5)	0.92 (0.67-1.25)	552 (90.2)	60 9.8)	1.28 (0.83-1.99)
Educational level						
Low/middle	773 (83.3)	155 (16.7)	1 [Reference]	594 (88.0)	81 (12.0)	1 [Reference]
High	425 (82.7)	89 (17.3)	1.40 (1.02-1.94)	374 (91.0)	37 (9.0)	1.02 (0.65-1.60)
Diabetes						
No	1139 (83.4)	227 (16.6)	1 [Reference]	926 (89.7)	106 (10.3)	1 [Reference]
Yes	48 (77.4)	14 (22.6)	2.34 (1.19-4.60)	34 (77.3)	10 (22.7)	2.22 (0.97-5.07)
Blood pressure ≥140/90 mm Hg						
No	801 (84.6)	146 (15.4)	1 [Reference]	764 (95.0)	40 (5.0)	1 [Reference]
Yes	408 (80.6)	98 (19.4)	0.91 (0.65-1.26)	212 (73.1)	78 (26.9)	6.82 (4.40-10.58)
Prescription of antihypertensive drug at baseline						
No	859 (85.5)	146 (14.5)	1 [Reference]	NA	NA	NA
Yes	350 (78.1)	98 (21.9)	1.63 (1.18-2.25)	NA	NA	NA
Prescription of lipid-lowering drug at baseline						
No	NA	NA	NA	902 (89.8)	103 (10.2)	1 [Reference]
Yes	NA	NA	NA	74 (83.1)	15 (16.9)	1.59 (0.81-3.13)
Serum cholesterol, mg/dL						
<193	314 (92.4)	26 (7.6)	1 [Reference]	231 (87.8)	32 (12.2)	1 [Reference]
193-250	693 (84.2)	130 (15.8)	2.02 (1.26-3.25)	518 (89.6)	60 (10.4)	0.65 (0.39-1.10)
>250	202 (69.7)	88 (30.3)	5.20 (3.09-8.76)	227 (89.7)	26 (10.3)	0.57 (0.31-1.08)
Year when participants were recruited to VIPVIZA^a^						
First year	410 (88.7)	52 (11.3)	1 [Reference]	308 (89.5)	36 (10.5)	1 [Reference]
Second year	343 (82.1)	75 17.9)	1.66 (1.10-2.51)	299 (91.4)	28 (8.6)	0.70 (0.40-1.23)
Third year	456 (79.6)	117 (20.4)	2.09 (1.42-3.07)	369 (87.2)	54 (12.8)	1.09 (0.66-1.79)
Vascular age^b^						
Green/yellow	345 (88.0)	47 (12.0)	1 [Reference]	279 (92.4)	23 (7.6)	1 [Reference]
Orange/red	864 (81.4)	197 (18.6)	1.57 (1.08-2.29)	697 (88.0)	95 (12.0)	1.38 (0.81-2.34)
Carotid plaque						
No	786 (91.4)	74 (8.6)	1 [Reference]	622 (92.7)	49 (7.3)	1 [Reference]
Yes	421 (71.2)	170 (28.8)	3.77 (2.73-5.22)	353 (83.8)	68 (16.2)	2.32 (1.48-3.64)

Abbreviations: cIMT, carotid intima media thickness; NA, not applicable.

SI conversion value: To convert low-density lipoprotein and total cholesterol to millimoles per liter, multiply by 0.259.

^a^ First year: April 2013-June 2014, second year: July 2014-June 2015, third year: July 2015-June 2016.

^b^ Vascular age was indicated by a gauge from green (cIMT in persons at least 10 years younger than their chronologic age) through yellow and orange to red (cIMT in persons at least 10 years older than their chronologic age).

For lipid-lowering drugs, the logistic regression models showed that having diabetes (OR, 2.52; 95% CI, 1.50-4.23), an antihypertensive drug at baseline (OR, 1.70; 95% CI, 1.30-2.23), or a higher level of total serum cholesterol (193-250 mg/dL: OR, 1.90; 95% CI, 1.29-2.81; >250 mg/dL: OR, 5.87; 95% CI, 3.86-8.94 compared with <193 mg/dL) increased the odds of a first prescription (Table 2). In the multivariable model for antihypertensive drugs, first prescriptions were associated with higher blood pressure (OR, 6.73; 95% CI, 5.00-9.05), diabetes (OR, 2.15; 95% CI, 1.17-3.93) or age 50 years compared with 40 years (OR, 1.98; 95% CI, 1.05-3.75). Prescription of lipid-lowering drugs increased over the study period, but there was no trend over time for antihypertensive drugs.

Prescriptions were renewed within 465 days after the ultrasonographic examination in 523 (91.8%) of the 570 participants with a baseline prescription for a lipid-lowering drug and 1233 (95.2%) of the 1295 participants with a baseline prescription for an antihypertensive drug. There were no significant differences between the intervention and control groups in prescription of either lipid-lowering drugs (control: 247 of 274 [90.1%] vs intervention: 276 of 296 [93.2%]; *P* = .18) or antihypertensive drugs (control: 614 of 640 [95.9%] vs intervention: 619 of 655 [94.5%]; *P* = .23) to participants with a baseline prescription for these agents.

## Discussion

Pictorial information about the presence of subclinical carotid atherosclerosis had a different effect on physician prescription of lipid-lowering drugs compared with antihypertensive drugs. Prescription of lipid-lowering but not antihypertensive drugs increased if participants and their physicians received pictorial information about the presence of carotid plaques and vascular age based on carotid ultrasonography findings (the intervention group) compared with those who did not receive any information (the control group). Prescription of lipid-lowering drugs was also more common among those with an increased risk of CVD (ie, diabetes, hypertension, or higher baseline levels of total cholesterol). Prescription of antihypertensive drugs was associated with blood pressure level, diabetes, and age. The presence of plaque was also associated with prescription of antihypertensive drugs, although the provision of pictorial information had no influence on the total prescription of antihypertensive drugs in the intervention group. Information about vascular age was associated with higher levels of prescription for lipid-lowering agents but not antihypertensive drugs (eAppendix 2 in Supplement 2). The difference in first prescription rates between lipid-lowering drugs and antihypertensive drugs reflects the advice provided by guidelines: antihypertensive treatment is recommended in patients with established CVD only if they have hypertension.^5^ Because there were few individuals with a baseline blood pressure greater than or equal to 140/90 mm Hg and no previous antihypertensive treatment (n = 579), the power to find a difference between the groups in the rate of new prescriptions of antihypertensive drugs was lower than for lipid-lowering drugs.

The VIPVIZA study was performed within the VIP, a population-based program for CVD prevention; hence, the study provides information on the feasibility of conducting such intervention in a real-world setting. All participants were followed up, and the outcome (prescription of drugs) was objectively measured in all participants. A lipid-lowering or antihypertensive drug is available by prescription only in Sweden. Our study included all prescriptions for lipid-lowering and antihypertensive drugs in the Region Västerbotten database.

After receiving the results, participants in the intervention group were contacted by telephone from a research nurse for support and clarification, but the participants or primary care physicians had to initiate another appointment or prescription if this action was considered important. The outcome of this study was physician prescribing behavior, but prescribing also depends on the participants´ inclination to seek health care and interest in using drugs.^15^ All study participants (intervention and control groups) were also VIP participants and hence had previously received risk factor information and a health dialogue aiming at health promotion. When needed according to guidelines, they also received a follow-up and physician evaluation regarding pharmacologic treatment. Thus, the only difference between the intervention and control groups was the pictorial presentation of carotid ultrasonography results.

The presence of plaque is an anatomical marker of atherosclerosis and estimates the probability of cardiovascular events in asymptomatic individuals.^16^ Individuals with plaques were, according to the current clinical guidelines,^5^ classified as having a very high risk for CVD and should, in most cases, be prescribed lipid-lowering drugs and, if their blood pressure is high, antihypertensive drugs. Information about whether participants had been offered treatment but declined was not available. Among participants not using drugs, there could have been an intention to try lifestyle changes instead of or before initiating drug treatment. The use of evidence-based drug treatments for prevention of CVD has also been low in other studies.^1,17^

Previous studies on the association between atherosclerosis detection and the prescription of drugs are mainly nonrandomized.^18,19,20^ In an observational study from the US on the outcomes of carotid ultrasonographic screening, physicians were more likely to prescribe lipid-lowering drugs but not antihypertensive drugs to participants with carotid plaques or cIMT greater than the 75th percentile.^18^ In a nonrandomized study from the UK, there was a significant increase in the use of antihypertensive but not lipid-lowering drugs in participants with plaque or cIMT greater than or equal to 1 mm.^19^ Furthermore, a meta-analysis suggested that identifying calcified coronary plaques increased the initiation of both lipid-lowering and antihypertensive drug treatment.^20^ None of these studies used pictorial images to inform participants about the ultrasonographic examination results.

### Limitations

This trial had limitations. Drugs prescribed outside health services funded by the Region were not included in our analysis; however, because the Region funds almost all health care in Västerbotten, this factor was not likely to have any significant influence on the results. We did not use information on the quantities prescribed, prescription duration, whether a prescription was withdrawn due, for example, to adverse drug reactions, or whether the prescribed drugs were actually used.

## Conclusions

To our knowledge, this is the first randomized clinical trial on the effect of pictorial information from carotid ultrasonographic screening on the prescription of lipid-lowering and antihypertensive drugs in individuals with subclinical atherosclerosis. The first prescription of lipid-lowering but not antihypertensive drugs was higher in the 465 days after the prescription if participants and their physicians were provided with pictorial information based on the ultrasonographic findings combined with a nurse-led follow-up call to the participants compared with no information about ultrasonography results.

## Notes

Supplement 1.
Trial Protocol and Statistical Analysis Plan

Supplement 2. **eAppendix.** Supplementary Information on the Ultrasound Examinations **eFigure 1.** Pictorial Information as Presented to Study Participants and Their Primary Care Physicians (Translated From Swedish) **eTable 1.** Operational Definitions of Variables **eFigure 2.** The CONSORT Flow Diagram **eTable 2.** Number of Subjects and Percentages With a First Prescription for Any Lipid-Lowering Drug Among Participants Without a Prescription at Baseline (n = 2962) in the VIPVIZA Study **eTable 3.** Number of Subjects and Percentages With a First Prescription for Any Antihypertensive Drug Among Participants Without a Prescription at Baseline (n = 2237) in the VIPVIZA Study

Supplement 3.
Data Sharing Statement
