# Supplementary material for: Prescription of Lipid-Lowering and Antihypertensive Drugs Following Pictorial Information About Subclinical Atherosclerosis: A Secondary Outcome of a Randomized Clinical Trial
Source: JAMA Netw Open. 2021 Aug 19;4(8):e2121683. doi: 10.1001/jamanetworkopen.2021.21683 (PMC8377571; doi:10.1001/jamanetworkopen.2021.21683)
Supplement: Supplement 1. — Trial Protocol and Statistical Analysis Plan [file jamanetwopen-e2121683-s001.pdf]

Visualization of Asymptomatic Atherosclerotic Disease for Optimum Cardiovascular Prevention: VIPVIZA – a Population-based RCT nested in Routine Care in Sweden

NCT01849575

Study Protocol Version 5.0

Visualization of Asymptomatic Atherosclerotic Disease for Optimum Cardiovascular Prevention: VIPVIZA – a Population-based RCT nested in Routine Care in Sweden

Study ID: VIPVIZA

Protocoll version: Version 4.0

Principal Investigator, PI: Ulf Näslund, Department of Public Health and clinical medicine, Umeå University.
Co-PI: Margareta Norberg, Department of Public Health and clinical medicine, Umeå University.

Start of the study:	2012-08-01
End of study:	2026-12-31

Visualization of Asymptomatic Atherosclerotic Disease for Optimum Cardiovascular Prevention: VIPVIZA – a Population-based RCT nested in Routine Care in Sweden

Study ID: VIPVIZA

Protokollversion: Version 4.0

Principal Investigator, PI: Ulf Näslund, Department of Public Health and clinical medicine, Umeå University.
Co-PI: Margareta Norberg, Department of Public Health and clinical medicine, Umeå University.

Start of the study: 2012-08-01

End of study: 2026-12-31

PROTOCOL AGREEMENT

PI and co-PI undertake to complete the study in accordance with protocols, and, referring to national and local regulations, in accordance with the Helsinki Declaration: Ethical Principles for Medical Research that Include People.
PI and co-PI agree, through written consent, to this Protocol and to fully participate and allow direct access to all documentation, including source data, for the relevant authorities.

The agreement above is signed

Signature: ______________________ Date: November 10 2017

Ulf Näslund, Professor

Signature: ______________________ Date: November 10 2017

Margareta Norberg, Associate professor

TABLE OF CONTENTS

1 Abbreviations and definitions.....7
2 Supplementary files.....8
3 Research team, contact information.....8
4 Summary..... 10
5 Background 12
5.1 Survey of the field and rationale 12
5.2 Hypotheses 13
5.3 Theoretical model.....13
6 Objective and aims..... 14
6.1 Objective.....14
6.2 Specific aims.....14
7 Endpoints..... 15
7.1 Primary endpoints (or effect variable?) 15
7.2 Secondary endpoints 15
8 Project description 15
8.1 Study design.....15
8.2 Time plan.....15
8.3 Setting and research environment.....16
8.4 Risk-benefit evaluation 16
8.5 Discontinuation of the study 17
8.6 Pilot study 17
9 Study plan, the main study.....17
9.1 Flowchart.....17
9.2 Visits.....18
9.2.1 Baseline visit, participation in VIP.....18
9.2.2 Baseline visit, ultrasound examination.....18
9.2.3 Follow-up after one year.....18
9.2.4 Follow-up after three years.....18
9.3 Activity scheme.....19
10 Study population.....20
10.1 Target group and invitation.....20
10.2 Selection criteria 20
10.2.1 Inklusion criteria 20
10.2.2 Exklusion criteria 20
11 Examinations and measurements 21
11.1 Demography, socio-economic and psychosocial data.....21
11.2 Life style anad health status.....21
11.3 Clinical risk factors for CVD 21
11.4 Psychometric data.....21
11.5 Ultrasound data.....22
11.6 Qualitative data 22
11.7 Biomarkers 22

11.8 Registry data 22
11.8.1 Pharmacological treatment....22
11.8.2 Socio-economic status....23
11.8.3 Cognitive characteristics....23
11.8.4 Clinical events.... 23
12 Intervention and control....23
12.1 Randomization and invitation to visits....23
12.2 Intervention group....23
12.2.1 Procedures....23
12.2.2 Image based pictorial information....26
12.3 Control group, procedures 27
13 Statistical analyses and considerations....27
13.1 Power and number of participants 27
13.2 Statistical analyses 28
13.2.1 Dataset and study population 28
13.2.2 Interim analyses 28
13.2.3 Summary of planned analyses 28
13.3 Planned drop-ut analysis....30
13.3.1 Non participants to participation....30
13.32 Drop outs at one year follow-up....30
13.3.3 Drop outs at three year follow-up....30
14 Study administration....31
14.1 Procedures for collection of Informed Consent....31
14.2 Definition of sorce data....31
14.3 Quality control....32
14.4 Quality assessment....32
14.5 End of the study....32
14.6 The VIPVIZA research data base and its management 32
14.7 Import of data to the database....33
14.8 Proposal application system and data handling 33
15 Ethical and legal aspects 34
15.1 Ethical considerations 34
15.2 Approval of the study....35
15.3 Major amendments....36
16 Funding of the study and insurances.... 36
17 References.... 37

1. Abbreviations and definitions

VIPIVZA	**VI**suali**Z**ation of asymptomatic **A**therosclerotic disease for optimum cardiovascular prevention – a pragmatic randomized controlled trial nested in the **V**ästerbotten **I**ntervention **P**rogram. The setting is highlighted by VIP positioned first in the acronym.
BMI	Body Mass Index
BMQ	Beliefs about Medicines Questionnaire
CIMT	Carotid artery Intima-Media Thickness
CVD	Cardiovascular disease
GCP	Good Clinical Practice
HDL	High density Lipoprotein
HbA1c	Hemoglobin A1c
LDL	Low Density Lipoprotein
KFC	The clinical research center at Umeå University Hospital [Swedish: Kliniskt Forsknings Center]
PCI	Percutaneous Coronary Intervention.
PTCA	Percutaneous transluminal coronary angioplasty
RCT	Randomized Controlled Trial
SCORE	Systematic Coronary Risk Evaluation
SP	Study participant in VIPVIZA
VIP	Västerbotten Intervention PRogramme

2. Supplementary files

Appendix 1 The Informed Consent form translated from Swedish to English.

Appendix 2 VIPVIZA diagnoses clinical events and causes of death

Appendix 3 VIPIVZA Database Description

Appendix 4 Statistical analyses plan, primary outcomes at one-year evaluation

Appendix 5 Monitoring plan

All documents in Swedish are available from co-PI Margareta Norberg. This include all applications to the Regional Ethical Board and the respective decisions, written information about the study to VIP-participants, information about VIPVIZA to district nurses and family physicians, written information to VIPVIZA-participants, questionnaires used in the study, the written report on ultrasound results and forms for postal information about results on CVD risk factors.

3. Research team, contact information

Role	Name, title, Tel	Institution/department/ address	Function in the study
**Principal Investigator (PI) / sponsor**	Ulf Näslund, MD, PhD<br>Prof<br>+46 70 514 6322	Umeå University, Department of public health and clinical medicine, Heart center, SE-90187 Umeå, Sweden	Principal Investigator/Sponsor<br>Steering group
**Co-PI/study director**	Margareta Norberg, MD, PhD<br>Associate prof<br>+46 70 187 1335	Umeå University, Department of public health and clinical medicine, Epidemiology and global health, SE-90187 Umeå, Sweden	Co-PI/Study director<br>Steering group
**Co-Investigators**	Per Wester, MD, PhD<br>Prof<br>Tel +46 70 328 0001	Umeå University, Department of public health and clinical medicine, Stroke Center, SE-90187 Umeå, Sweden	Researcher<br>Steering group
	Christer Grönlund, PhD<br>title<br>tel +46 70 330 4544	Umeå University, Department of Radiation sciences, SE-90187 Umeå, Sweden	Researcher<br>Steering group
	Bernt Lindahl, MD, PhD<br>Associate prof<br>Tel +46 70 2737 805	Ume Umeå University, Department of public health and clinical medicine, Behavioral Medicine, SE-90187 Umeå, Sweden å	Researcher<br>Steering group
	Joakim Rocklöv<br>Associate prof<br>Tel +46 70 636 1635	Umeå University, Department of public health and clinical medicine, Epidemiology and global health, SE-90187 Umeå, Sweden	Statistician, researcher<br>Steering group
	Patrik Wennberg, MD, PhD<br>Associate prof<br>Tel +46 73 832 8819	Ume Umeå University, Department of public health and clinical medicine, Family Medicine, SE-90187 Umeå, Sweden å	Researcher<br>Steering group
	Stefan K Nilsson, PhD<br>Tel +46 70 578 1768	Umeå University, , Department of Medical Biosciences, Unit of Physiological chemistry , SE-90 187 Umeå, Sweden	Researcher<br>Steering group
	Bertil Lindahl<br>Prof<br>+46 701429580	Uppsala university, UCR-Uppsala Clinical Research Center, SE-75183 Uppsala	Researcher<br>Steering group

	Bo Carlberg, MD, PhD<br>Ass prof<br>+46 70 577 5883	Umeå University, Department of public health and clinical medicine, Medicine, SE-90187 Umeå, Sweden	Researcher
	Steven Nordin, PhD<br>Prof<br>Tel +46 70 609 6006	Umeå University, Department of psychology, SE-90187 Umeå, Sweden	Researcher
	Maria Nordin, PhD<br>Associate prof<br>+46 90 7869924	Umeå University, Department of psychology, SE-90187 Umeå, Sweden	Researcher
	Maria Sjölander, PhD<br>Post doc<br>Tel +46 73 736 4804	Umeå University, Department of public health and clinical medicine, SE-90 187 Umeå	Researcher
	Gunilla Olivecrona MD, PhD<br>Prof<br>+46 70 5480262	Umeå University, Department of Medical Biosciences, Unit of Physiological chemistry, SE-90 187 Umeå	Researcher
	Per Lindqvist<br>Associate prof<br>+46 707601502	Umeå University, Department of Surgery and perioperative sciences, Clinical physiology, Heart Centre, SE-90 187 Umeå, Sweden	Researcher
	Helene Johansson,PhD,<br>Physioteraphist<br>+46 73 025 5173	Umeå University, Department of public health and clinical medicine, Epidemiology and global health, SE-90187 Umeå, Sweden	Researcher
	Eva Fhärm,<br>MD, PhD<br>+46 70 351 5166	Umeå University, Department of public health and clinical medicine, Family Medicine, SE-90187 Umeå, Sweden å	Researcher
	Kristina Lindvall, PhD,<br>Dietician<br>Tel +47 70 584 5952	Umeå University, Department of public health and clinical medicine, Epidemiology and global health, SE-90187 Umeå, Sweden	Researcher
	Nawi Ng, Md, PhD<br>Prof<br>Tel +46 72 540 3851	Umeå University, Department of public health and clinical medicine, Epidemiology and global health, SE-90187 Umeå, Sweden	Researcher
	Davide Vanoli MD, PhD<br>MD, PhD<br>+46 070 396 1161	Umeå University, Department of public health and clinical medicine, Heart center, SE-90187 Umeå, Sweden	Researcher
**Publication manager**	Rachel Nicoll, PhD<br>Research assistant<br>Tel +44 7917 787350	Umeå University, Department of public health and clinical medicine, SE-90187 Umeå, Sweden	Publication manager, researcher
**Data base manager**	Wolfgang Lohr<br><br>+46 90 7865205	Umeå University, Department of public health and clinical medicine, Epidemioogy and global health, SE-90187 Umeå, Sweden	Data base manager
**Doctoral Students**	Anna Lundgren MD<br>Emma Nyman<br>Fredrik Landfors<br>Oleg Kovrov<br>Ka Chung Tsan		
**Monitor**	Clinical Trial Unit	Unit for clinical research, Västerbotten county council, SE-908 Umeå	

4. Summary

Scientific title:	Visualization of Asymptomatic Atherosclerotic Disease for Optimum Cardiovascular Prevention: VIPVIZA – a Population-based RCT nested in Routine Care in Sweden
Principal Investigator<br>Study director	Ulf Näslund<br>Margareta Norberg
Study period	2012-08-01–2026-12-31
Hypotheses and aims	VIPVIZA has three distinct, but complementary hypotheses:<br>Pictorial representation of asymptomatic atherosclerotic disease, assessed with carotid ultrasound, has the potential to identify individuals at high risk of CVD with higher precision than conventional risk factor-based statistical models.<br>The clear understanding of an image of arterial disease during an early asymptomatic phase is of superior value in optimizing adherence to clinical preventive management for both physicians and patients, Literally, the English quotation is ‘A picture speaks a thousand words’.<br>The resulting preventive management will improve control of premature CVD.<br>*Specific aims*<br>To assess the prevalence of asymptomatic atherosclerotic disease in men and women through identification of carotid plaques and measurement of CIMT, and to relate plaques and CIMT to clinically estimated CVD risk factors.<br>To assess the impact of visualization of atherosclerosis on quality of life, preventive measures, risk factor control and progress of atherosclerotic disease over the course of three years.<br>To explore the impact of visualization of atherosclerosis, as a low-intensive preventive intervention, on physicians´ and patients´ risk perception, communication and attitudes to CVD prevention.<br>To evaluate how individuals’ health literacy and coping strategies relate to CVD risk at baseline, attitudes to screening, risk perception and preventive measures.<br>To investigate novel biomarkers in relation to CIMT and plaques at baseline, changes in conventional CVD risk markers and lifestyle, and progression of atherosclerosis.<br>To assess the impact of visualization of atherosclerosis by carotid ultrasound on premature CVD morbidity and mortality over the course of 5 and 10 years.
Primary outcome	SCORE and Framingham risk scores

Secondary outcomes	CVD risk factors, lifestyle, ultrasound results (CIMT, presence of plaque, quantitative and qualitative plaque parameters, presence and degree of significant stenosis), prescriptions and purchases of medications (for the treatment of hypertension, dyslipidemia and diabetes), biochemical markers, and the clinical endpoints myocardial infarction, stroke, revascularization procedures and mortality (all-cause and CVD-specific).
Study design	A pragmatic randomized controlled trial nested within routine clinical care
Study population	Subjects aged 40, 50 or 60 years, invited as part of their VIP participation within Västerbotten primary healthcare.<br>No:3700
Follow-up period	10 years from first ultrasound examination
Inclusion criteria	• age=40 years and first-degree relative with history of CVD at age <60 years<br>• age=50 years and at least one of the following: first-degree relative with history of CVD at age <60 years, smoking, diabetes, hypertension, S-LDL cholesterol ≥4.5 mmol/L, abdominal obesity<br>• age=60 years<br>Individuals are included in the study only once, at whichever qualifying age point is achieved first.
Statistical methods	The differences in primary outcomes (SCORE risk and Framingham Risk Score) at one year between the treatment groups will be measured and analyzed using t-test. Differences in changes of SCORE and FRS from baseline to 1-year follow-up will be analyzed with regression methods to identify predictors of the changes in the scores.<br>Survival curves will be established using Kaplan-Meier curves for CVD mortality and morbidity at 5 and 10 years of follow up comparing the intervention to the control group. Predictive Cox Proportional Hazard models will be developed to estimate the overall predictive ability of CIMT, plaque and risk factors on CVD morbidity and mortality at 5 and 10 years.<br>Health economic modelling studies will be performed for evaluation of cost-effectiveness. Interviews with participants and physicians will be analyzed using qualitative content analysis.
Evaluation criteria	Primary and secondary outcomes will be evaluated according to Intention-to-treat methodology

5. Background

5.1Survey of the field and rationale for the trial

Primary prevention of cardiovascular disease (CVD) often fails due to the low precision of the conventional risk scores, and poor adherence among practitioners and patients to evidence-based prevention guidelines. Moreover, these risk scores focus on high-risk individuals only, despite 60-70% of all CVD events occurring among individuals at low or intermediate risk for CVD (1). VIPVIZA takes a different approach from current practice for the prevention of CVD. Instead of being based solely on indirect risk factors, this project evaluates the atherosclerotic disease itself while it still is silent, providing improved assessment, communication and perception of the CVD risk and higher motivation for prevention.

The evidence for effective modification of risk factors by lifestyle change and pharmacological treatment is well established. Despite this, suboptimal adherence to CVD prevention guidelines among practitioners and patients leads to poor risk factor control in both primary and secondary prevention (2-4). Contributory factors include poor communication about the CVD risk by the physician, inaccurate risk perception and lack of awareness of the weak association between the actual estimated and perceived CVD risk among patients(5, 6). The risk message is usually communicated verbally or numerically (7), while potentially more effective visual tools are seldom used. In addition to the format and framing of the message, the perceived risk also depends on social and cultural factors (8).

Moreover, for the success of risk management, the extent of the patient's behavioral change is crucially dependent on their psychological characteristics. The individual's ability to cope with stressful situations (coping strategy) (9), trust in their own capability to change behaviour (self-efficacy) (10), level of positive expectations in life (dispositional optimism) (11), and health literacy (capacity to understand health information and make appropriate related health decisions) (12) are important. Rational choices due to increased risk awareness often fail, and knowledge about a risk, on its own is rarely enough for sustainable behavioural change. These aspects are usually ignored in the field of primary prevention (13). For CVD prevention programs to be effective, a more person-centred approach towards the individual's clinical and behavioural change may need to be implemented (14), and in order to make use of existing evidence-based methods to the fullest extent, both the physicians' and individuals' adherence to existing CVD prevention guidelines should be targeted.

An alternative strategy to evaluate and improve CVD risk communication would be to directly view in pictorial form the degree of atherosclerotic disease before symptoms develop, rather than to indirectly evaluate risk factors of atherosclerosis (15-17). This is achieved with ultrasonography of medium sized arteries with assessment of carotid artery intima-media thickness (CIMT) and existing atherosclerotic plaques, which have a clearly higher predictive power for plaques. Pharmacological

and lifestyle interventions have the potential to slow down or even reverse the progress of ultrasound-assessed atherosclerosis (18, 19), but results on the contributory impact from image-based information on adherence to prevention are inconsistent (20, 21).

Therefore, large-scale randomized controlled trials (RCTs) targeting low and intermediate-risk adults with a focus on assessing the impact of viewing an image of existing silent disease on the stratification and communication of CVD risk to physicians and patients, as well as on major clinical outcomes, should be prioritized (1, 2, 21).

5.2 Hypotheses

VIPVIZA has three distinct, but complementary hypotheses:

- Direct viewing of a pictorial representation of asymptomatic atherosclerotic disease, assessed with carotid ultrasound, has the potential to identify individuals at high risk of CVD with higher precision than conventional risk factor-based statistical models.
- The clear understanding of an image of arterial disease during an early asymptomatic phase is of superior value in optimizing adherence to clinical preventive management for both physicians and patients: ”A picture speaks a thousand words”.
- The resulting preventive management will improve the control of premature CVD.

5.3 Theoretical model

Social, genetic, psychological and behavioral mechanisms impact the subclinical molecular processes that manifestas clinical risk factors and the subclinical morphologic atherosclerotic process, and eventually in clinical cardiovascular disease events or death.

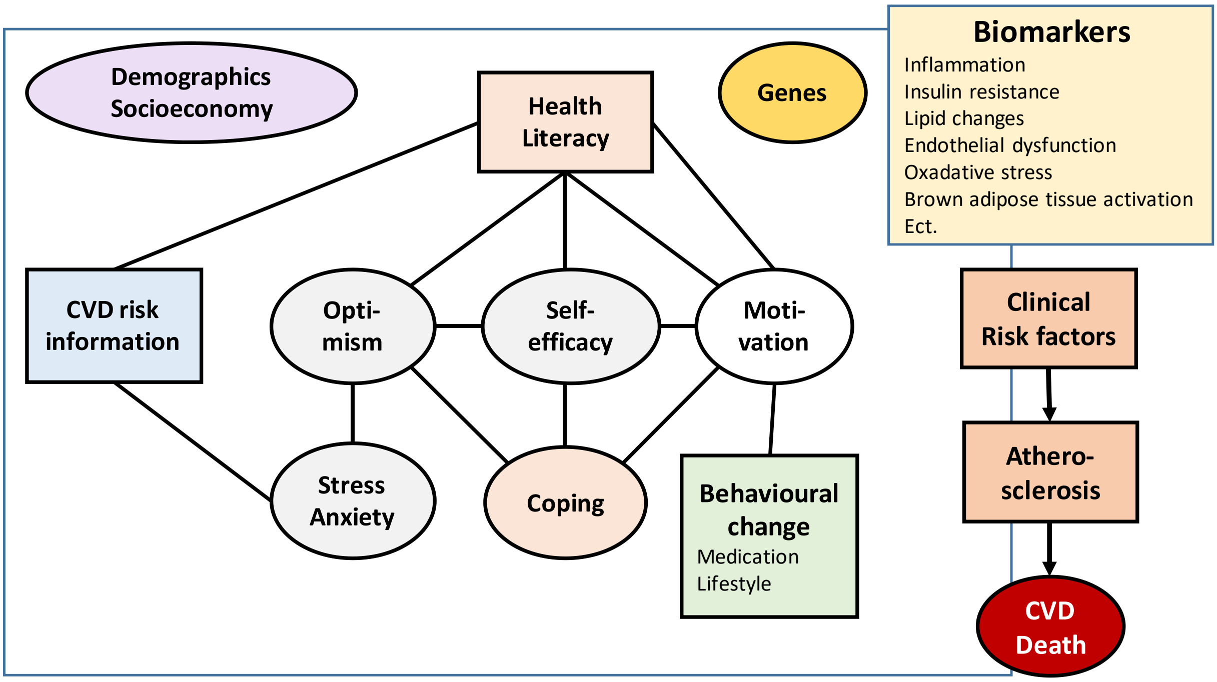

6. Objective and aims

6.1 Objective

To assess the impact of a visual image and pictorial report, seen and discussed by both physician and patient, for improving guideline adherence and patient perception and understanding of the CVD risk and consequent motivation for prevention. The extent of the impact is assessed by differences between randomization groups in Framingham Risk Score and SCORE as well as changes in these scores after one and three years. Differences between groups will also be evaluated by risk factor assessment and a further scan after three years to determine disease progression, and a comparison of the prevalence of acute events and mortality at five and 10 years.

6.2 Specific aims

1. To assess the prevalence of asymptomatic atherosclerotic disease in men and women through identification of carotid plaques and measurement of CIMT, and to relate plaques and CIMT to clinically estimated CVD risk factors.
2. To assess the impact of pictorial representations of atherosclerosis on quality of life, preventive measures, risk factor control and progress of atherosclerotic disease over the course of three years.
3. To explore the impact of pictorial representations of atherosclerosis, as a low-intensive preventive intervention, on physicians´ and patients´ risk perception, communication and attitudes to CVD prevention.
4. To evaluate how individuals' health literacy and coping strategies relate to CVD risk at baseline, attitudes to screening, risk perception and preventive measures.
5. To investigate biomarkers in relation to CIMT and plaques at baseline, changes in conventional CVD risk markers and lifestyle, and progression of atherosclerosis.
6. To assess the impact of the pictorial representation of atherosclerosis by carotid ultrasound on premature CVD morbidity and mortality over the course of 5 and 10 years.

7. Endpoints

7.1 Primary endpoints:

7.1.1 SCORE (22)

7.1.2 Framingham risk score (23)

7.2 Secondary endpoints

7.2.1 CVD risk factors (blood pressure, serum cholesterol, LDL, HDL, triglycerides, fasting glucose, HbA1c)

7.2.2 Lifestyle (physical activity, tobacco use, alcohol use, eating habits)

7.2.3 Ultrasound results (CIMT, presence of plaque, quantitative and qualitative plaque parameters, presence and degree of significant stenosis)

7.2.4 Pharmacological treatments of hypertension, dyslipidemia and diabetes, prescriptions and purchases

7.2.5 Biochemical biomarkers

7.2.6 Clinical endpoints: Hospitalizations due to CVD, diagnostic and therapeutic revascularization procedures (PTCA, PCI, stent) regarding atherosclerotic disease and deaths (all-cause and CVD-specific), as specified in Appendix 2: VIPVIZA diagnoses clinical events and causes of deaths

7.2.7 Quality of life.

8 Project description

8.1 Study design The design is a pragmatic randomized controlled trial (24, 25).

8.2 Time plan A summary of the time plan for participants' visits in the study as well as for interviews and acquisition of register data from the National Board of Health and Welfare is shown in the figure below.

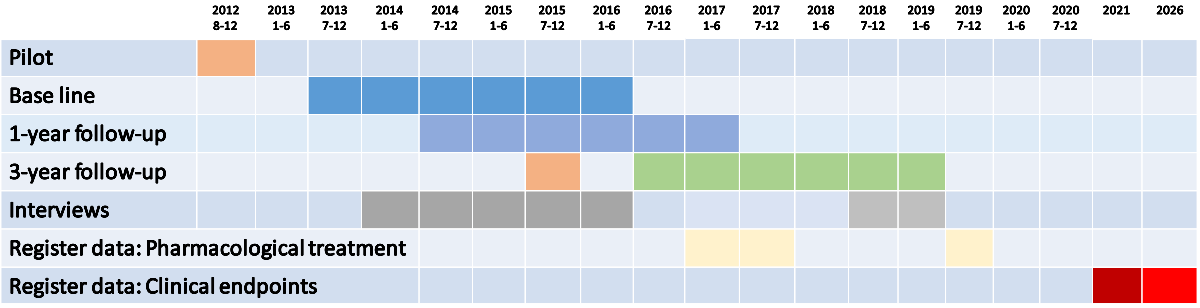

8.3 Setting and research environment

VIPVIZA is integrated in the Västerbotten Intervention Program (VIP) (26), which is a population-based CVD screening and prevention program in the County of Västerbotten, Sweden (population 265,000). Since the 1990s, VIP has provided health surveys for all county inhabitants during the year in which they turn 40, 50 and 60 years (n=6500-7000/year), comprising CVD risk factor screening and individual promotion of healthy lifestyle and pharmacological CVD prevention.

To date, over 170,000 health surveys have been performed. Participation rates during recent years were around 60-70% with only small social selection bias (27). VIP is one of the largest long-term CVD prevention programs in the world. It provides a unique arena for pragmatic studies of population-based CVD risk intervention

The ultrasound examinations in VIPVIZA are performed at the hospitals in three cities/towns (Umeå, Skellefteå, Lycksele), and in remote rural areas at primary health care centres. Risk factor measurements and questionnaires at follow-up are carried out for participants in Umeå at the Clinical Research Centre at Umeå University Hospital, and for participants in the rest of the county at their local primary health care center.

8.4 Risk-benefit evaluation for the individual

The intervention in VIPVIZA is the provision of pictorial information about the presence and extent of the individual's atherosclerosis from their ultrasound results. This is considered to be a low-intensity intervention in comparison to interventions with pharmacological drugs or surgical procedures. The ultrasound examination cannot cause any harm, physical discomfort or risk.

As with all screening which targets a healthy population, it is a dilemma whether or not to inform asymptomatic individuals of silent disease, in this case an ongoing atherosclerotic process with increased risk of future CVD. The presence of atherosclerosis can be perceived by the subject as more serious than just an increased level of risk markers, and may therefore result in anxiety. In order to avoid unjustified concerns, all persons in the intervention group receive a telephone call from a research nurse and, if necessary, a doctor in charge, to give in-depth and balanced information about the ultrasound results. This conversation is conducted according to the methodology of motivational interviewing and also aims to increase awareness of the possibility of reducing the individual's risk by means of their own preventive measures. This is expected to alleviate anxiety and increase motivation to follow the recommendations for preventive treatment. According to the study hypotheses, this is expected to benefit the individual due to risk reduction and a more healthy lifestyle is also expected to bring benefits through increased well-being and quality of life. Similarly, subjects without ongoing atherosclerotic disease will be able to avoid unjustified concerns that their risk factors, if any, may be contributing to an active disease process.

All individuals with severe carotid stenosis will be excluded from the study and are referred directly to the Stroke Center for assessment and treatment. This may potentially be life-saving for these people, and may, to some extent, mitigate the fact that no information is provided to half the group until 3 years after they enter the study, even if small/moderate changes are detected.

8.5 Discontinuation of the study

Individuals at high risk of stroke, defined as severe carotid stenosis, i.e. stenosis >50% of the lumen, are excluded from the study, informed of the results and referred to the Stroke Center, University Hospital, Umeå, with treatment in accordance with current guidelines for this group.

8.6 Pilot study

A pilot study with 95 participants was performed September-October 2012. The study organisation worked well and protocols and procedures were further developed and showed good feasibility, although randomization to intervention and control groups was not carried out. In order to optimize understanding of atherosclerosis as a process and the accurate perception of the ultrasound result, the information was calibrated according to participants' experiences and suggestions, collected through questionnaires and interviews. After 3 years, complete data on risk factors from 81 pilot participants was collected, and favorable changes regarding LDL-cholesterol, triglycerides and fasting glucose were observed, with the addition of favorable weight changes among women only.

9. Study plan, the main study

9.1 Flow chart

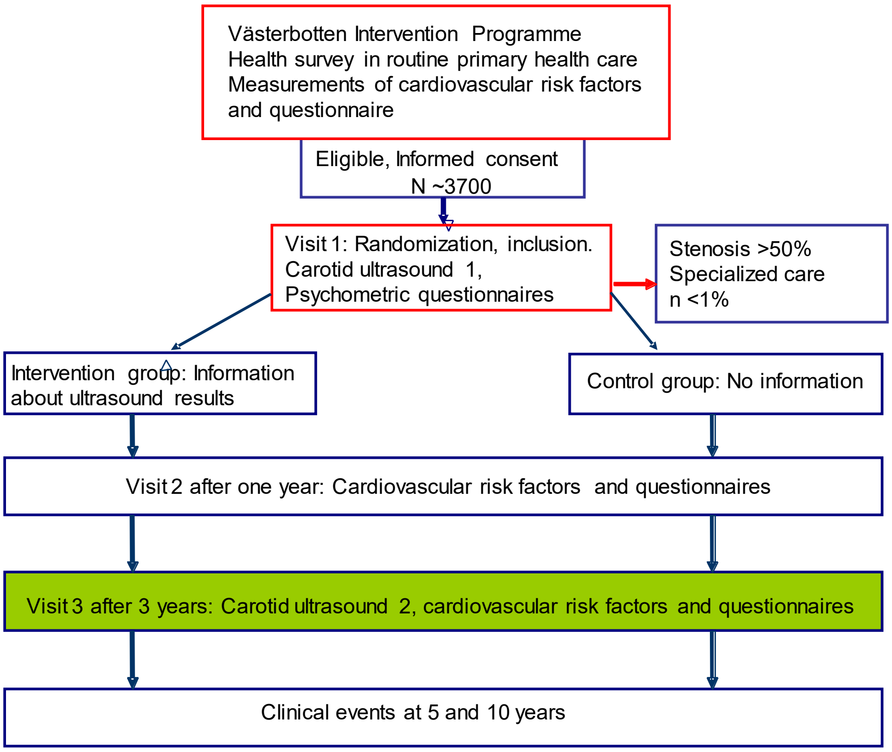

9.2 Visits

9.2.1 Baseline visit, participation in VIP. Data collection: demographic, socio-economic, clinical risk factors for CVD, and lifestyle. This data is used in an individual health dialogue with a trained district nurse at the primary health care center. Applying the methodology of Motivational Interviewing, this session aims at health promotion and CVD prevention. A pictorial tool, the “Star profile” (Figure below), is used to facilitate the individual’s understanding of how his/her lifestyle is linked to CVD risk factors.

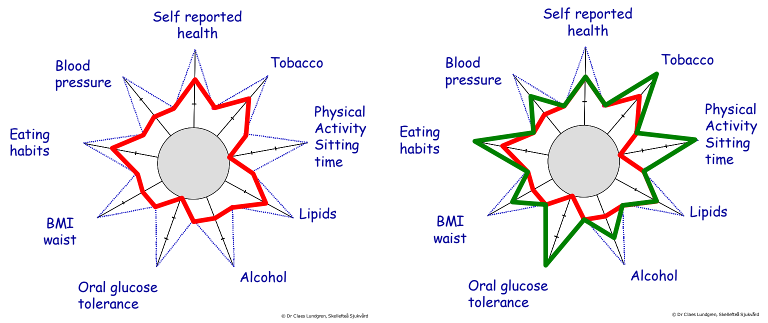

When requested, VIP-participants are also recommended follow-up visits or referrals to the family physician for pharmacological treatment according to guidelines. Samples of blood are frozen andstored at the Biobank, Umeå University Hospital.

VIP participants who are eligible for participation in VIPVIZA are given written and verbal information about the VIPVIZA study and are invited to the study at the occasion of their participation in VIP at their primary health care centre. The district nurses who conduct the health dialogues also sign that the study information is given and collect the informed consent forms and send them to research nurses at KFC. The research nurses register all forms. Appendix 1: The Informed Consent form translated from Swedish to English

9.2.2 Baseline visit with ultrasound examination. Study participants are randomized into two equal groups, intervention and control, following a computer-generated randomization list. They then complete psychometric questionnaires, questions about self-rated risk of CVD and health-specific self-efficacy, and undergo the first ultrasound examination of both carotids. Written information about results are sent to those in the intervention group and their respective family physician in primary care.

9.2.3 Follow-up one year after the ultrasound examination. Measurement of clinical risk factors for CVD, lifestyle, self-reported pharmacological treatment, self-rated health, risk of CVD and health-specific self-efficacy. Framingham Risk Score and Score are estimated.

9.2.4 Follow-up three years after the ultrasound examination Data collection: clinical risk factors for CVD, completion of lifestyle questionnaire, psychometric questionnaires, questions about self-rated risk of CVD and health-specific self-efficacy. Participants also undergo the second ultrasound examination of both carotids. Samples of blood are drawn, frozen and stored at the

Medical Biobank, Umeå University Hospital. Written information about ultrasound results are sent to all SPs and their respective family physicians in primary care.

9.3 Activity scheme

	Base line		Follow-up			Action defined
	Visit 1 VIP exam	Visit 2 Ultrasound	Visit 3 1 year	Visit 4 3 years	5 and 10 years	
Informed consent	X					9.2.1, 11.1, 12.1, 14.1
Clinical risk markers	X		X	X		9.2.1, 9.2.3, 9.2.4, 11.3, 12.2, 12.3
Lifestyle habits	X		X	X		9.2.1, 9.2.3, 9.2.4, 11.2, 12.2.1, 12.3
Demographic socioeconomyic and psychosocial factors						9.2.1, 11.1, 11.8.2
Ultrasound examination		X		X		9.2.2, 9.2.4, 11.5, 12.2.1, 12.2.2, 12.3
Psychometric questionnaires		X		X		9.2.2, 9.2.4, 11.4, 11.8.3
Self-rated health, risk of CVD and health-specific self-efficacy		X	X	X		9.2.1, 9.2.2, 9.2.3, 9.2.4, 11.4, 12.2.1
Interviews with GPs		X				11.6, 12.2.1
Interviews with study participants		X		X		11.6, 12.2.1
Pharmacological treatment	X		X	X		9.2.1, 9.2.3, 9.2.4, 11.2, 11.8.1
Biomarkers	X			X		9.2.1, 9.2.4, 11.7
Hospitalizations registry data					X	11.8.2, 11.8.4
Deaths registry data					X	11.8.2, 11.8.4

10 Study population

10.1 Target group and invitation

The study focuses on subjects at intermediate risk of CVD. Based on VIP data from 2012, around 60% of all VIP participants of 40, 50 and 60 years of age, i.e. n= 4000/year, was estimated to be at moderate risk of CVD due to having at least one conventional CVD risk factor (see below for inclusion criteria). Since VIP is on-going continuously in primary care, inclusion can continue until the desired number of SPs is reached.

VIP participants who are eligible for participation in VIPVIZA are given written and verbal information about the VIPVIZA study and are invited to the study at the occasion of their participation in VIP at their primary health care centre. The district nurses who conduct the health dialogues also sign that the study information is given and collect the informed consent forms and send them to research nurses at KFC. The research nurses register all forms (baseline visit performed in the VIP).

10.2 Selection criteria

10.2.1 **Inclusion criteria** were based on 1/ the aim of targeting subjects at intermediate risk of CVD, and 2/established clinical criteria (smoking, diabetes, hypertension, abdominal obesity) or evaluation of levels and distribution of clinical CVD risk markers (S-LDL) in the 2011 VIP population. In this population, the cut-off for the 4th quartile among 50 years old men and women was 4.4 mmol/L and 4.0 mmol/L, respectively, while 9% and 12% respectively had S-LDL concentrations ≤2.5 mmol/L, and 81% and 88% respectively had S-LDL concentrations ≤4.5 mmol/L.

- age=40 and a history of CVD at age <60 years among first-degree relative(s)
- age=50 years and at least one of the following: a history of CVD at age <60 years among first-degree relative(s), smoking, diabetes, hypertension, S-LDL-cholesterol ≥4.5 mmol/L, abdominal obesity defined by waist >88cm for women and >102 cm for men
- age=60 years

Individuals are included in the study only once, at whichever qualifying age point is achieved first.

10.2.2 Exclusion criteria

Significant stenosis as defined as >50% luminal narrowing of the investigated carotid arteries according to vascular ultrasound. These individuals are informed about results irrespective of randomization

status, are excluded from the study and referred to the Stroke Center, Umeå University Hospital, for further evaluations and treatment.

11 Examinations and measurements

11.1 Demography, socio-economic and psychosocial data

From the baseline VIP questionnaire: Age, sex, civil status, education, family history of premature CVD and diabetes, social network and support according to the International schedule on social interaction (Swedish version) (28) and work stress (according to the Karasek-Theorell demand/control model) (29).

11.2 Lifestyle and health status

From VIP data at base line and at 1- and 3-year follow-up visits: Questionnaire data including self-reported physical activity, sitting time, tobacco habits, alcohol (AUDIT questionnaire), diet (food-frequency questionnaire, except at the 1-year visit), self-rated health, self-reported (known) diabetes as well as blood pressure and lipid-lowering pharmacological treatment. At 3-year follow-up, the Swedish version of the Beliefs about Medicines Questionnaire (BMQ) (30, 31)was also added from March 2017.

11.3 Clinical risk factors

At baseline, 1- and 3-years. Measurements:

- Anthropometrics: Waist is measured on the skin at the point mid-way between the last palpable rib and the iliac crest, using a non-elastic measuring tape; height and weight are measured in light clothing without shoes using calibrated scales and stadiometers.
- Systolic and diastolic blood pressure is measured twice with a calibrated digital blood pressure gauge at the precision of 2 mm after 5 minutes rest with the subject in a sitting position. The mean value of the two systolic and diastolic measurements are recorded.
- Lipids: Blood samples for serum-cholesterol, serum-LDL, serum-HDL, serum-triglycerides are drawn after an overnight fast and sent to the department of Clinical Chemistry at the nearest local hospital and analyzed with standard clinical biochemical methods. The three hospital labs in the county are all quality assured.
- Glucose status: Within the VIP (26), at baseline after an overnight fast, an oral glucose tolerance test is carried out according to WHO standards, with measurements of capillary fasting plasma glucose and 2-hour plasma glucose (32). At 1- and 3-year follow-up, capillary fasting plasma glucose is measured using HemoCue® Glucose analyzer (HemoCue AB, Ängelholm, Sweden).

11.4 Psychometric data

Psychometric data will be utilized as moderators and/or mediators of the intervention effect on primary and secondary outcomes, and also as outcomes at 3-year examination. At baseline and 3-year examination: Health literacy (33), Coping strategies (Brief Cope) (34), General Self-Efficacy (35) , Anxiety and Depression (HAD) (36), Optimism/Pessimism (LOT-R) (37). At base-line, 1- and 3-year follow-up there are also questions about self-rated health (5 alternatives) (38), self-rated risk of CVD and health-specific self-efficacy (ability to reduce the CVD risk through preventive actions) using a

VAS scale 0-10. In a subset of the study population, the Newest Vital Sign test on health literacy (39) was added to the questionnaire at 1-year follow-up from January 2017 until completion of 1-year visits in August 2017.

11.5 Ultrasound data

IMT variables measured at specific positions and angles on both sides of the arteria carotis. Since there is no international consensus on which specific IMT measurement to use optimally, in particular in early stage atherosclerosis, a separate explorative analyses will be performed on this issue. Vascular age is calculated based on IMT and related to IMT values among subjects matched for sex and age in the Atherosclerosis Risk In Communities study population (ARIC) (40). This reference study was selected due to similarity with risk factor patterns in the VIP population. Plaque is defined according to the Mannheim Consensus (41). The presence of plaque on both sides of the carotid artery, plaque area and plaque texture are recorded.

11.6 Qualitative data

Telephone interviews with around 25 purposively sampled participants from the intervention group (based on age, sex, ultrasonography results) are conducted within four weeks of receiving the baseline ultrasound information, as well as at 3-year follow-up. Interviews will also be performed with practitioners, who have received at least five different patients' ultrasound results, to explore how viewing the ultrasound results affects physicians´ perceptions of their patients' risk, the communication with patients and attitudes to treatment of CVD risk factors.

11.7 Biomarkers

Plasma samples are collected at baseline for the entire study population and in a subsample of SPs living in Umeå and surrounding municipalities. These samples are centrifuged and stored at -80º C at the Medical Biobank, Umeå University Hospital, to be analyzed at the 3-year follow-up for hypothesized CVD biomarker. Examples ares:

- The end product of oxidized LDL detected via autoantibodies against distinct apoB peptides and its prospective correlation to current atherosclerotic burden (42, 43).
- Bile acid receptors: the farsenoid xenobiotic receptor (FXR) and the G-protein coupled receptor TGR5 (44) and their longitudinal role related to CVD.
- Lipoprotein lipase (LPL) activity (45) to assess whether substrate differences for LPL translates into predictive risk changes (Lookene et al. in preparation).
- Metabolomics, particularly lipidomics: (Umeå Plant Science Centre, SciLifeLab, and Uppsala Clinical Research Centre, Sweden)

11.8 Registry data

11.8.1 Pharmacological treatment: At baseline, 1 and 3 years: Prescriptions retrieved from medical records in the Västerbotten County Council digital system registries. At 1 and 3 years: purchases of pharmacological products from the Statistics on Pharmaceuticals at the National Board of Health and Welfare.

11.8.2 Socio-economic status: Income and highest attained educational level from Statistics Sweden.

11.8.3 Cognitive characteristics at age 18 (males only): The Conscripts register at the National Archives Database.

11.8.4 Clinical events: From data retrieved from registers at the National Board of Health and Welfare at 5 and 10 years after the last ultrasound examination: Hospitalizations due to cardiovascular morbidity and diagnostic and therapeutic procedures and mortality (all-cause and CVD-specific), according to the International Classification of Diagnoses ICD10, as specified in Appendix 2 VIPVIZA diagnoses clinical events.

12 Intervention and control

12.1 Randomization and invitations to visit.

VIP participants who are eligible for participation in VIPVIZA are given written and verbal information about the VIPVIZA study and are invited to the study at the occasion of their participation in VIP at their primary health care centre. The district nurses who conduct the health dialogues also sign that the study information is given and collect the informed consent forms and send them to research nurses at KFC. The research nurses register all forms.

SPs are then randomized to two equal groups (intervention and control group) before the first ultrasound examination. The randomization list is generated prior to the study through simulation from a uniform probability distribution. At this stage a unique identification code is assigned to each SP. Invitation with appointment times for the baseline visit is sent by mail to both groups by the research nurses who manage the study at KFC.

12.2 Intervention group

12.2.1 Procedures

- Baseline: Research nurses send invitation letters with an appointment time for the baseline ultrasound examination by mail to those who gave informed consent to participation in VIPVIZA. One reminder letter with a new appointment time is sent to non-attenders. At this visit, participants answer the psychometric questionnaires and undergo the baseline ultrasound examination. Ultrasound examinations are performed by sonographers specifically trained in carotid ultrasound techniques (Biomedical Scientists) from the Department of Clinical Physiology, Heart Centre, Umeå University Hospital. Examinations are undertaken in hospitals in three towns in Västerbotten county (Umeå, Skellefteå and Lycksele), and at the local primary healthcare centres in rural areas. A portable automatic carotid ultrasound equipment (CardioHealth Station®, Panasonic Healthcare Corporation of North America, Newark, NJ, USA) is used. A standardized protocol according to current guidelines is applied for the examinations (46, 47). The angle of insonation is automatically provided by the system and is recorded. Measurements of CIMT (max, min and mean values)

are automatically obtained (41). The same ultrasound machine is used during the entire study period.

Within two weeks of the ultrasound examination, information about the carotid ultrasound results are sent by post to the participant, see 12.2.2. At the same time, exactly the same information is sent to their respective primary care physician. After an additional 2-4 weeks participants receive a follow-up phone call by a research nurse in order to ascertain whether the information was accurately understood and to reassure the participant and give additional information if needed.

- Qualitative interviews with a subgroup of around 20 SPs within the intervention group concerning their experiences and reactions to the information about the ultrasound results.
- Qualitative interviews with General Practitioners who have received at least five written reports from VIPVIZA regarding their patients.
- At 6 months, participants receive a reminder in the form of a letter with the identical information about the ultrasound results and very brief reminder about possible preventive measures.
- At 9 months, participants receive a letter with information about proceedings in the study and reminder about the 1-year follow-up.
- At 1 year, participants receive an invitation to the follow-up visit, with an appointment time at KFC. This is sent by research nurses to participants living in Umeå and surrounding municipalities. The information is also sent to the health care centers outside the Umeå area, where VIPIVIZA participants are eligible for the 1-year follow-up. Nurses at these health care centers conduct the 1-year visits for their VIPIVZA participants. This visit includes measurement of risk factors and a questionnaire including self-rated health, health-related quality of life (RAND 36), prescribed medication for hypertension and dyslipidemia, tobacco and alcohol use, physical activity, usual portion sizes as an indicator of possible changes of eating habits, and self-rated risk of CVD as well as possibilities to reduce this risk, i.e. health-specific self-efficacy.

  The results regarding risk factor measurements are provided on a form that has been approved by the Steering Group and also includes recommendations for preventive measures depending on the levels of risk factors. This form is sent by post by the research nurses at KFC and district nurses at health care centers.
  In the intervention group, the questionnaire which was sent during the spring semester 2017 to the last 500 participants before completion of the 1-year visits, includes questions concerning how participants perceived and recalled the pictorial ultrasound information and the phone call received at baseline
- At 2 years, a letter is sent with information about proceedings in the study and a reminder about the 3-year follow-up.
- At 2.5 years, a letter is sent with repeated information about the atherosclerotic process, as well as general information regarding healthy eating.

- At the 3-year follow-up: Invitations and appointment time for the ultrasound examination, risk factor measurement and questionnaire completion is sent by post to participants living in Umea and surrounding municipalities by research nurses at KFC; all examinations are conducted at KFC. Participants outside this area are also invited for ultrasound examinations undertaken in hospitals in the three towns in Västerbotten County (Umeå, Skellefteå and Lycksele), and at the local primary health care centres in rural areas. Invitations are sent only once, with no reminder following non-attendance. . Research nurses also inform the health care centers outside the Umeå area when it is time to invite participants to the 3-year visit, although this visit is managed by the health care centers, including risk factor measurements and questionnaire completion. At this time point, the intervention group also answer questions about how they perceived the pictorial information and phone call received at baseline concerning the ultrasound results.

Results of clinical risk factor measurements and the completed questionnaires are sent to KFC and the research nurses give feedback to participants using a predefined form similar to the form used at the 1-year follow-up.

Within two weeks of the carotid ultrasound examination, information about the results are sent by post to the participant, see 12.2.2. At the same time, exactly the same information is sent to their respective primary care physician. After an additional 2-6 weeks, participants whose ultrasound results indicated CIMT values higher than expected according to age and sex (red gauge) or with plaques (red), receive a follow-up phone call by a research nurse in order to ascertain whether the information is accurately understood and to reassure the participant and give additional information if needed. Participants without plaques (green) or vascular age similar or lower than expected (green or yellow/orange gauge) are informed by a standard letter including the contact information for research nurses, in case of questions or concerns.

Qualitative interviews are conducted with a few SPs, regarding their experience of receiving the second ultrasound report and how they interpreted this in relation to any life style modification carried out, including taking prescribed medications, following the first ultrasound examination.

12.2.2 Image-based pictorial information

This information was developed during the pilot phase. The original report from the CardioHealth station was tested and refined in communication with VIPVIZA pilot participants, with patients at the clinic of Behavioral Medicine, who had a different educational status and who had lifestyle related metabolic diseases. The research nurses' experiences from discussions of the results with participants were also considered. The team of researchers compiled the collected impressions and developed the written information, aiming to provide easy and accurate understanding of the scanning result and concerning atherosclerosis as a dynamic process that can be modified by lifestyle changes and pharmacological treatment. A

technician adjusted the print scripts accordingly, so that the approved information is automatically generated by the CardioHealth station, including both graphics and text.

The information includes the following:

- The carotid intima media thickness is presented as vascular agee with graphic presentations of atherosclerosis highlighted in color against normal vascular age patterns as a gauge, proceeding from a green sector, through yellow and orange to a red sector to illustrate the percentiles 1-25, 26–50, 51–75 and 76–100, respectively. Since no data are available from VIPVIZA's source population, the Atherosclerosis Risk In Communities study population (ARIC) (40) was selected as the reference population for calculation of vascular age by sex and age due to the similarity with the VIP population of risk factor patterns.
- Plaque formation shown as a traffic light for each side of the carotid artery, with a green circle for 'no plaque detected' and a red circle for 'plaque detected'.
- A stylized picture of the participant's own ultrasound image showing vascular age as a colored line and plaques as a red mark.
- Brief written information about atherosclerosis as a dynamic process, which is modifiable by a healthy lifestyle and pharmacological treatments for hypertension, dyslipidemia and diabetes.

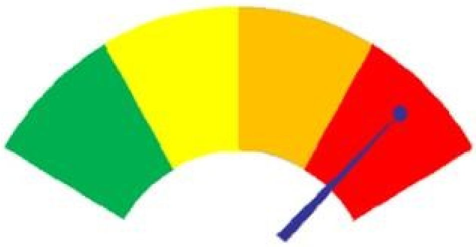

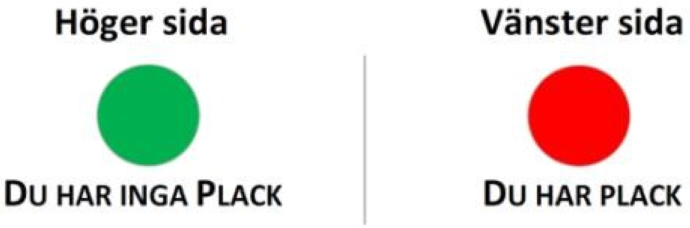

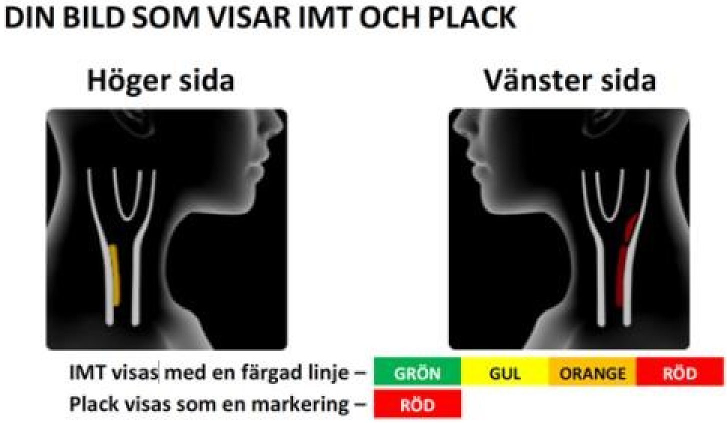

12.3 Control group procedures

- Baseline: Identical to procedures for the intervention group, except that no information about ultrasound results are sent to SPs and their respective GPs, and there are no qualitative interviews.
- At 1-year: Identical to follow-up procedure for the intervention group, except that there is no sub-study regarding perceptions about the baseline ultrasound report.
- At 3-years: Identical to follow-up procedures for the intervention group, except that there are no questions regarding perceptions about the baseline ultrasound report and the phone call and no qualitative interviews.

13 Statistical analyses and considerations

13.1 Power and number of study participants

Calculations were performed to estimate the sample size needed to detect a clinically significant difference between the control group and the intervention group with sufficient power regarding change from baseline to 1-year follow-up in the primary clinical outcome variables, Framingham Risk Score and SCORE, as well as for S-LDL and total cholesterol, systolic blood pressure. Regarding possible detectable change of CIMT from baseline to 3-year follow-up, the available literature reported a mean change of 0.010±05 mm/year from the MESA study (48) and in a meta-analysis the progression was 0.000-0.023/year for mean maximal IMT of the common carotid artery (49). For evaluating the effects of the various CIMT variables on the sample-size calculation for a trial, we used a pooled common CIMT progression rate of 0.0147 mm/y (0.044 mm/3 y) with the corresponding SD. For the mean maximum progression rates, we used an estimate of 0.0176 mm/y (50).

Since no data was available from the current population, we hypothesized that a change of CIMT of 0.02 mm would be theoretically possible to detect having standardized protocols for IMT assessment including multiple angles of insonation, anatomic landmarks and automated edge detection software technology as was also recommended in a state of the art paper (15). As the design utilizes randomization, the groups were assumed comparable at baseline, thus no further adjustment for background factors was considered. As a lower limit for power we used a threshold of 0.8, and for significance level we used 0.05. These calculations were based on standard deviations on conventional risk factors derived from VIP 2012 and on CIMT from the Tromsö study (51). The power calculations revealed that an initial total number of 3500 participants with a drop-out rate of 15 % during the study would be sufficient. The limiting factor demanding the largest group size to show a hypothesized detectable effect was CIMT. Thus, the sample size was decided based on that.

Variabel	Precision	Power	SD	Relevant change Population level	Sample size per group	Minimum detectable change if n=1500/group
SBP (mmHg)	0.05	0.8	16	2	1500	1.7
Serum Cholesterol (mmol/L)	0.05	0.8	0.5	0.5	1500	0.05
LDL (mmol/L)	0.05	0.8	0.5	0.5	1500	0.05
CIMT (mm)	0.05	0.8	0.2	0.02	1500	0.02
SCORE	0.05	0.8	1.40	0.5	1500	0.143
Framingham Risk Score	0.05	0.8	6.60	1	1500	0.7

13.2 Statistical analyses

13.2.1 Dataset and study population

All subjects participating in the study will be included in the analyses. For the drop-out analyses, non-participants (those who did not consent to participation or failed to attend the first ultrasound examination) will be included in the analyses of selection bias for participation, but this evaluation will be based on only a restricted number of variables (sex, age, education, clinical CVD risk factors and lifestyle).

13.2.2 Interim analyses

No interim analysis is planned. Any eventual differences in deaths and clinical events such as myocardial infarction, stroke and revascularisation procedures cannot be expected to be identified and analyzed statistically before three years of follow-up in this middle-aged, low/intermediate risk population. Furthermore, since they participate in the VIP, the control population (not informed) receives primary prevention actions according to guidelines and to a relatively high extent in comparison with most other counties in Sweden and most other countries globally.

13.2.3 Summary of planned analyses

The differences in primary outcomes (SCORE risk and Framingham Risk Score) at one year between the treatment groups will be measured and analysed using t-test, for a detailed statistical analyses plan see Appendix 4. As the group sizes are large (more than thousand), it is valid to assume normal distribution of the error around the mean value estimates in both the intervention and the control group. A two-sided p-value <0.05 will be regarded as statistically significant. Complementary to the absolute difference in the Score and the statistical testing of hypotheses, Cohen´s effect size will be derived for the difference in mean outcome values divided by a pooled standard deviation of the Score between the 2 groups.

In sensitivity analyses, we will conduct a linear regression covariate-adjusted analysis. We will use the continuous form of the primary outcomes (SCORE risk and Framingham Risk Score) as outcome in the

regression analysis. We will assess the effects of the intervention independently for each of the outcome measures. As many of the baseline co-variates (including sex and age) are related to the prognosis of outcomes, adjustment of pre-defined covariates in the regression analysis might yield in adjusted coefficients which are slightly further from 1.

We will also analyze the changes of SCORE and FRS from baseline to 1-year follow-up and use regression methods to identify predictors of the changes in the scores.

Since patients are recruited into the study during three years from mid-2013 to mid-2016, any change in physicians' prescribing behaviour over the course of the study will be evaluated by comparing prescriptions issued for lipid-lowering and anti-hypertensive medication to participants recruited during the first, second and third years.

Similar statistical methods will be applied for evaluation of the three-year results. At that time point also changes in plaque distributions and IMT variables including differences between intervention and control group will be evaluated.

In long-term, adding risk communication using visualisation of carotid ultrasound results will be compared to routine CVD risk assessment and control within the primary health care setting in hospitalisation due to stroke, myocardial infarctions and revascularisations (at 5-year and 10-year) and reducing overall mortality and cause-specific mortality due to myocardial infarctions and stroke (at 5-year and 10-year) compared to.

Simple survival curves will be established using Kaplan-Meier curves for CVD mortality and morbidity at 5 and 10 years of follow up comparing the intervention to the control group. Thereafter, predictive Cox Proportional Hazard models will be developed to estimate the overall predictive ability of CIMT, plaque and risk factors on CVD morbidity and mortality at 5 and 10 years in the control group and in the intervention group, taking into account time at risk for disease and deaths. Receiver Operating Characteristic Curves (ROC) will be calculated by estimating the Area Under the Curve (AUC) to ensure the highest possible sensitivity and specificity of the predictions. Confirmatory and exploratory statistical modelling will establish relationships between the intervention and intermediate and mediating factors and variables' relative contribution to explain changes in the outcome variables.

Since participants are recruited into the study during the three years from mid-2013 to mid-2016, any change in physicians' prescribing behaviour over the course of the study will be evaluated by comparing prescriptions issued for lipid-lowering and anti-hypertensive medications among participants recruited during the first, second and third years.

As a basis for how to build regression models Causal diagrams (directed acyclic graphs - DAGs) will be established and causal mediation analysis will be undertaken to illustrate the impact of social, gender, psychological and environmental factors by biomedical risk indices on CVD morbidity and mortality.

Health economic modelling studies will be performed for evaluation of cost-effectiveness. Interviews with participants and physicians will be transcribed verbatim and analyzed using Qualitative Content Analysis (52).

13.3 Planned drop-out analyses

13.3.1 Non-participants vs participants

This category includes all individuals who were informed about VIPVIZA at the occasion of participation in the VIP and who did not consent (ticked 'NO' on the form) to participation in VIPVIZA, as well as subjects who initially consented to participate but withdrew their consent, either actively by phone or mail to the research nurses, or passively, through failing to attend the baseline ultrasound examination. One reminder to those who fail to attend is sent with a new appointment time for the baseline ultrasound examination but if they still fail to attend, they are categorized as a non-participant. Aggregated data on the group of non-participants will be compared with the included study participants regarding CVD risk markers and lifestyle as well as educational level and civil status. In this analysis, published data on participation and selection to the VIP will also be considered (27).

13.3.2 Drop-outs at the 1-year evaluation

At one year, the study population will be further reduced as a result of exclusions due to significant carotid stenosis (10.2.2), deaths and migration out from the county. Drop-outs will be defined as active withdrawal of consent to participation or failure to attend the 1-year follow-up evaluation after postal invitation and one reminder. Drop-outs will be compared to the other participants regarding risk factors, lifestyle, level of education, and intervention group status at baseline.

13.3.3 Drop-outs at the 3-year evaluation:

SPs will be invited to the 3-year examination irrespective of participation status at the 1-year follow-up, provided they were not excluded according to 10.2.2 or have withdrawn their consent). Therefore, drop-outs at Year 3 will be defined as active withdrawal following the 1 year examination or failure to attend the 3-year follow-up after postal invitation and one reminder. Drop-outs will be compared to the other participants regarding risk factors, lifestyle and educational level as well as intervention group status at baseline.

14 Study administration

14.1 Procedures for collection of informed consent

At the occasion of participation in the VIP, subjects are given written information about VIPVIZA, including the form for informed consent. Those who fulfill the inclusion criteria are informed verbally by the VIP nurse, who also collects the forms, both from those who consent to and those who decline to participate in the study. These forms are sent to the research nurses at KFC, who insert the data from those who consent into the electronic CRF document. Subjects who do not consent to participation in the study are listed separately for analysis of selection bias using aggregated data and a restricted number of variables.

14.2 Definition of source data

There are several different sources of data in VIPVIZA. See also below section Data Management.

Sources for VIPVIZA data comprise:

- VIP data: The VIP questionnaires are sent from healthcare centers to the Medical Biobank, Umeå University Hospital, where they are registered and optically read by ITS, Umeå University. The electronic data are sent to the VIP database at the Unit of Epidemiology and Global Health, Umeå University. The questionnaires are stored at the Medical Biobank, Umeå University Hospital.
- The Case Report Form (CRF) contains information about randomization, as well as administrative information about visits, exclusions and drop-outs. The CRF is electronic and managed by the research nurses and located within the Västerbotten County Council's computer system.
- All questionnaires answered by participants at base line, 1-year and 3-year visits are kept in files at the Clinical Research Center under a code for each participant. After completion of all visits in the study (June 2019) they will be stored at the Unit of Epidemiology and Global Health during the entire study period in a safe archive.
- Clinical risk markers at 1- and 3-year visits are written on the front page of the lifestyle questionnaire.
- Ultrasound data are registered and stored in the CardioHealth Station.
- Pharmacological prescriptions: Stored in the electronic medical records at the participants' respective primary health care centers within the Västerbotten County Council.
- Interviews: Audiotapes taken at the occasion for interviews [should we say something about secure storage?]
- Register data: The pharmaceutical register, the Statistics on inpatient diseases in Sweden, and the Statistics on causes of death are stored at the National Board of Health and Welfare. The Conscripts register is stored at the National Archives Database. Statistics Sweden (Educational attainment and income) [are we missing something here?]

14.3 Quality control

The study is quality-controlled based on protocols and data collection on site to verify that:

- That data is reliable, accurate and complete
- That the safety and rights of all involved in the study are protected
- That the study has been conducted in accordance with current protocols and study agreements as well as GCP and all applicable government requirements.

An external monitor is selected at the Clinical Trial Unit at Västerbotten County Council. The monitor is not involved in the study.

The monitor has the necessary education, and the scientific and/or clinical knowledge necessary to monitor the trial adequately.

The Principal Investigator approves that the monitor has direct access to all relevant documentation.

Monitoring takes place in accordance with current GCP during and after the study. If any violation or negligence is discovered, the Principal Investigator shall be notified of corrective and/or preventive measures. See Appendix 5 Monitoring plan.

14.4 Quality assessment

It is the responsibility of the principal Investigator to ensure that a) collected data is checked, complete, signed and dated, b) that the study is conducted according to the protocol, to study agreements, to GCP and obligations from authorities, bearing in mind and that regulatory control/audits/inspections could be made at any time during or after closure of the study c) ensure that co-investigators and departments participating in the study approve and assist in any kind of inspection.

14.5 End of the study

The study will be formally closed on Dec 31 2026. Study visits and data collection from participants will be finalized in June 2019 or when all 3-year visits are completed. From that time point, SPs will be followed through registers for an additional 10 years. Since there is no intervention other than providing information about silent atherosclerosis to all participants at baseline and at 3-year follow-up, which qualifies as clinical prevention according to guidelines for CVD preventions, any premature termination before Dec 31 2026 will not be applicable.

14.6 The VIPVIZA research database and its management

All data will be entered into the VIPVIZA research database, which is managed by a trained database manager. The database and the database management is described in detail in Appendix 3: VIPVIZA Database Description. This document describes the original data, source files and the structured data and their content, purpose and physical storage including hardware encryption and back-up. It also

describes the variable catalogue and proposal application system. The pass word is only known by the data manager and the data can only be accessed by the data manager. The pass word is kept in a sealed envelope in a safe at the Department of Public health and clinical medicine. In order to link data from external sources to the VIPVIZA database repeatedly and during the whole study period, personal identification numbers are retained in the data base.

The key code will be retained for 10 years until register data on clinical events are retrieved and then destroyed, or if the follow-up is terminated earlier, the key code will be destroyed in the same way. All personal data will be processed in accordance with the Personal Data Act (1998: 204) and the study participants are entitled to receive information on the data recorded in the study annually free of charge and to have any errors corrected. The exception to this is base-line ultrasound results for individuals in the control group.

The files containing consent forms, questionnaires, as well as copies of the ultrasound report for the intervention group, will be archived at the Department of Public Health and Clinical Medicine.

14.7 Import of questionnaire data, risk factor data, CRF and ultrasound data to the database

Questionnaire data and risk factor data are entered into an access file on two laptops that are used for this sole purpose. The database manager creates the access file and also imports the data from the access files, as well as data from the CRFs and the ultrasound data on IMT and plaque to the VIPVIZA database.

14.8 Proposal application system and data handling

Researchers do not have access to the database. Researchers who are affiliated to VIPVIZA have access to an internal webpage for VIPVIZA. On this page, available data sets and variables are listed, including aggregated information, but no individual data. The web page also offers functionality for developing and sharing research proposals. Every authorized researcher can create and upload a proposal using a standardized form. External researchers may also apply for data if they collaborate with at least one member of the steering group. In such cases, external researchers will get access to the internal web page to follow the proposal routines regarding applications and approvals. Data without personal identification numbers will be exported to the researcher by the data manager after approval from the VIPVIZA steering group. and results will be reported only at an aggregated level. No researcher will be able to identify any participant, and therefore publications based on the study do not cause any ethical dilemma.

15 Ethical and legal aspects of the study

15.1 Ethical considerations

The study will be conducted according to the ethical principles based on the Helsinki Declaration, GCP, national regulatory mandatory instructions and this study protocol.
Both written and oral information to all participants in the study declares that participation in the study is voluntary and can be withdrawn at any time without any consequences for their ordinary health care.

Study participants will be carefully informed in a standardized way to ensure full and adequate communication, including information about randomization to an intervention or control group and treatments within primary care, prescribed according to guidelines about cardiovascular risk factors and preventive treatments, irrespective of whether information about the vascular ultrasound is provided or not. The signed consent from participants also includes information about the monitoring of the study and access to their confidential medical records at the County Council of Västerbotten, as well as, when appropriate, medical records from private primary health care centers, and register data from national registers. Specific written consent applies to storage of data and blood samples at the Biobank. The participants can at any time communicate questions to research nurses and can, without any payment, obtain information about their individual data in the study and to revise any incorrect data.

The issue of not providing information about silent atherosclerosis to half of the study population was thoroughly discussed with the Regional Ethical Board. It was judged to be acceptable, because 1/ individuals with severe carotid stenosis (<1% in the pilot study) are excluded and promptly referred for expert care (through potentially life- or function-saving measures), and 2/ there are no specific treatments for mild-moderate atherosclerotic disease other than risk factor control, according to guidelines. Risk factor control will be given irrespective of whether or not the ultrasound results are disclosed to participants.
The ultrasound message was extensively tested during the pilot study 2012 to ensure that easily understandable information is reported, that is accurately interpreted. This was carried out among out-patients with intermediate risk of CVD at the clinic of Behavioural Medicine, among patients with history of a CVD event at Heart Centre, as well as among the pilot participants.

During the study, any negative reaction, such as anxiety due to image results, is balanced through the provision of additional information and supportive counselling by research nurses in a phone call to each participant. This issue is also specifically in focus in the qualitative sub-studies and its investigation is included in the objectives of the study.

It is possible and hypothesized that the compliance to guidelines for prevention of CVD among physicians at health care centers will increase as a result of the information about ultrasound results in the intervention group. This is not considered an ethical problem, since there are no studies

demonstrating improved prognosis by using imaging techniques among subjects without signs of high-degree flow-limiting stenosis of the carotid arteries, and no study participants are excluded from prevention measures according to the current CVD prevention guidelines for health care. Previous studies of imaging techniques in health screening for cardiovascular disease suggest improved risk stratification. Therefore, from an ethical perspective it is important to investigate new pathways towards improved prevention and to discard old inefficient methods.

The study is integrated into an environment with direct links to ordinary health care, with its routines for follow up and prevention, including rapid direct referrals if pathological conditions (requiring further diagnostic or therapeutic interventions) are detected. The study is performed with stable research conditions for all study participants

There is no selection of participants in the VIP. All citizens in the county have the same opportunity to be invited the year they are 40, 50 and 60 years old. The study has no negative effects for the health care of any citizen or group of citizens. Once invited to VIP, clear information is given that the health screening is combined with advice, motivational information and measures to improve long-term health. VIPVIZA adheres fully to all the intentions of VIP.
Altogether, the eventual improved risk stratification and risk communication to physicians and study participants outweighs any potential negative effect of the study. There is no risk for harm with the direct procedure for vascular ultrasound.

15.2 Approval of the study

The application was submitted to the Regional Ethics Examination Board in Umeå, December 13 2011 and was approved February 7 2012. Documented approval to participate in study activities according to the study protocol and informed consent forms was obtained from authorities of the County Council of Västerbotten, representing the participating primary health care centers and hospital clinics, as well as from managers of private primary health care centers. These documents were attached to the application to the Regional Ethical Board (Appendix 1). Applications for amendment concerning all significant changes to the protocol thereafter have been approved by the Regional Ethics Committee. The significant amendments are:

15.3 Major amendments

- Repeated risk factor measurements and questionnaires about lifestyle at a one-year follow-up (added visit) as well as at the 3-year follow-up.
- A questionnaire to all SPs concerning important determinants of perceived risk of CVD and performance of preventive lifestyle modifications.
- Interviews with primary care physicians concerning their perceptions and reactions to the baseline ultrasound information.
- Repeated ultrasound information to the intervention group 6 months after the baseline examination.
- The psychometric questionnaires added to the 3-year follow-up.
- Qualitative interviews at 3 years among SPs in the intervention group.
- Collection of samples of blood to be frozen and stored in the Medical Biobank, Umeå University Hospital at the 3-year visit.
- To keep the personal identification number in the database to allow for linkage with register data from different sources at different time points during the whole study period until collection of data on clinical events and deaths after 10 years. The personal ID is not available to researchers, only to the database manager.
- The Beliefs about Medicines Questionnaire added to the 3-year questionnaire.
- Extended ultrasound examinations, that are necessary in cases where the CardioHealth station does not provide sufficient technological quality to allow a definite evaluation of potential significant stenosis information. This includes contrast enhancement and advanced ultrasound imaging to evaluate plaque texture.
- Psychometric data from the Conscripts register and income and education from Statistics Sweden as well as data on air-borne pollutants.

16 Funding of the study and insurances

VIPIVZA is supported by the Swedish Research Council Dnr 521-2013-2708 and Dnr 2016-02891, Västerbottens County council ALFVLL-2988001, VLL-583721 and ALFVLL-643391, the Swedish Medical Society SLS-405351 and -503111, the Northern county councils VISARENORR 465621,561592 and 741711, the Heart and Lung Foundation Dnr 20150369 and 20170481, SKANDIA insurance company Risk Hälsa, the Heart Foundation of Northern Sweden, Riksförbundet Hjärt-Lung, , STROKE- the national association, the Foundation for Stroke Research in Northern Sweden, AFA Insurance and an unconditional donation from Carl Bennet Ltd, Sweden. The funders have no role in the design, methods, subject recruitment, data collections, analysis or preparation of manuscripts in the project. The views expressed in this study protcol are those of theresearchers and not those of the funders.

The Patient Injury Act applies to all patients and subjects participating in research treated in Swedish health care. The Patient Insurance as well as the Pharmaceutical Insurance also applies to the study participants in this study.

17 References

1. Polonsky TS, Greenland P. CVD screening in low-risk, asymptomatic adults: clinical trials needed. Nature reviews Cardiology. 2012;9(10):599-604.
2. Kolandaivelu K, Leiden BB, O'Gara PT, Bhatt DL. Non-adherence to cardiovascular medications. Eur Heart J. 2014;35(46):3267-76.
3. Karbach U, Schubert I, Hagemeister J, Ernstmann N, Pfaff H, Hopp HW. Physicians' knowledge of and compliance with guidelines: an exploratory study in cardiovascular diseases. Deutsches Arzteblatt international. 2011;108(5):61-9.
4. De Backer G, De Bacquer D, Ryden L, Kotseva K, Gaita D, Georgiev B, et al. Lifestyle and risk factor management in people at high cardiovascular risk from Bulgaria, Croatia, Poland, Romania and the United Kingdom who participated in both the EUROASPIRE III and IV primary care surveys. European journal of preventive cardiology. 2016;23(15):1618-27.
5. Safeer RS, Cooke CE, Keenan J. The impact of health literacy on cardiovascular disease. Vascular health and risk management. 2006;2(4):457-64.
6. Claassen L, Henneman L, Nijpels G, Dekker J, Marteau T, Timmermans D. Causal beliefs and perceptions of risk for diabetes and cardiovascular disease, The Netherlands, 2007. Preventing chronic disease. 2011;8(6):A130.
7. Neuner-Jehle S, Senn O, Wegwarth O, Rosemann T, Steurer J. How do family physicians communicate about cardiovascular risk? Frequencies and determinants of different communication formats. BMC family practice. 2011;12:15.
8. Fagerlin A, Ubel PA, Smith DM, Zikmund-Fisher BJ. Making numbers matter: present and future research in risk communication. American journal of health behavior. 2007;31 Suppl 1:S47-56.
9. Svensson T, Inoue M, Sawada N, Yamagishi K, Charvat H, Saito I, et al. Coping strategies and risk of cardiovascular disease incidence and mortality: the Japan Public Health Center-based prospective Study. Eur Heart J. 2016;37(11):890-9.
10. Luszczynska A, Scholz U, Schwarzer R. The general self-efficacy scale: multicultural validation studies. The Journal of psychology. 2005;139(5):439-57.
11. Carver CS, Scheier MF, Segerstrom SC. Optimism. Clinical psychology review. 2010;30(7):879-89.
12. Schulz PJ, Nakamoto K. Health literacy and patient empowerment in health communication: the importance of separating conjoined twins. Patient education and counseling. 2013;90(1):4-11.
13. Mauskop A, Borden WB. Predictors of statin adherence. Curr Cardiol Rep. 2011;13(6):553-8.
14. Arbab-Zadeh A, Fuster V. The myth of the "vulnerable plaque": transitioning from a focus on individual lesions to atherosclerotic disease burden for coronary artery disease risk assessment. J Am Coll Cardiol. 2015;65(8):846-55.
15. Naqvi TZ, Lee MS. Carotid intima-media thickness and plaque in cardiovascular risk assessment. JACC Cardiovascular imaging. 2014;7(10):1025-38.
16. Baber U, Mehran R, Sartori S, Schoos MM, Sillesen H, Muntendam P, et al. Prevalence, impact, and predictive value of detecting subclinical coronary and carotid atherosclerosis in asymptomatic adults: the BioImage study. J Am Coll Cardiol. 2015;65(11):1065-74.
17. Nicolaides A, Panayiotou AG. Screening for Atherosclerotic Cardiovascular Risk Using Ultrasound. J Am Coll Cardiol. 2016;67(11):1275-7.

18. Sala-Vila A, Romero-Mamani ES, Gilabert R, Nunez I, de la Torre R, Corella D, et al. Changes in ultrasound-assessed carotid intima-media thickness and plaque with a Mediterranean diet: a substudy of the PREDIMED trial. Arteriosclerosis, thrombosis, and vascular biology. 2014;34(2):439-45.
19. Riccioni G, Cipollone F, Santovito D, Scotti L, D'Orazio N, Mezzetti A, et al. Effect of 2-year treatment with low-dose rosuvastatin on intima-media thickness in hypercholesterolemic subjects with asymptomatic carotid artery disease. Expert Opin Pharmacother. 2011;12(17):2599-604.
20. Kalia NK, Miller LG, Nasir K, Blumenthal RS, Agrawal N, Budoff MJ. Visualizing coronary calcium is associated with improvements in adherence to statin therapy. Atherosclerosis. 2006;185(2):394-9.
21. Rodondi N, Collet TH, Nanchen D, Locatelli I, Depairon M, Aujesky D, et al. Impact of carotid plaque screening on smoking cessation and other cardiovascular risk factors: a randomized controlled trial. Arch Intern Med. 2012;172(4):344-52.
22. Conroy RM, Pyorala K, Fitzgerald AP, Sans S, Menotti A, De Backer G, et al. Estimation of ten-year risk of fatal cardiovascular disease in Europe: the SCORE project. Eur Heart J. 2003;24(11):987-1003.
23. D'Agostino RB, Sr., Vasan RS, Pencina MJ, Wolf PA, Cobain M, Massaro JM, et al. General cardiovascular risk profile for use in primary care: the Framingham Heart Study. Circulation. 2008;117(6):743-53.
24. Zwarenstein M, Treweek S, Gagnier JJ, Altman DG, Tunis S, Haynes B, et al. Improving the reporting of pragmatic trials: an extension of the CONSORT statement. BMJ. 2008;337:a2390.
25. Califf RM, Sugarman J. Exploring the ethical and regulatory issues in pragmatic clinical trials. Clin Trials. 2015;12(5):436-41.
26. Norberg M, Wall S, Boman K, Weinehall L. The Västerbotten Intervention Programme. Background, design and implications. Global health action. 2010;3:4643.
27. Norberg M, Blomstedt Y, Lonnberg G, Nystrom L, Stenlund H, Wall S, et al. Community participation and sustainability--evidence over 25 years in the Vasterbotten Intervention Programme. Global health action. 2012;5:1-9.
28. Henderson S, Duncan-Jones P, Byrne DG, Scott R. Measuring social relationships. The Interview Schedule for Social Interaction. Psychol Med. 1980;10(4):723-34.
29. Theorell T, Karasek RA. Current issues relating to psychosocial job strain and cardiovascular disease research. J Occup Health Psychol. 1996;1(1):9-26.
30. Horne R, Weinman J, Hankins M. The beliefs about medicines questionnaire: the development and evaluation of a new method for assessing the cognitive representation of medication. Psychol Health. 1999;14:1-24.
31. Horne R, Frost S, Hankins M, Wright S. “In the eye of the beholder”: Pharmacy students have more positive perceptions of medicines than students of other disciplines. Int J Pharm Pract. 2001;9:85-9.
32. WHO. Definition, Diagnosis and Classification of Diabetes Mellitus and its complications. report of a WHO consultation. 1999.
33. Chew LD, Griffin JM, Partin MR, Noorbaloochi S, Grill JP, Snyder A, et al. Validation of screening questions for limited health literacy in a large VA outpatient population. J Gen Intern Med. 2008;23(5):561-6.
34. Carver CS. You want to measure coping but your protocol's too long: consider the brief COPE. International journal of behavioral medicine. 1997;4(1):92-100.
35. Love J, Moore CD, Hensing G. Validation of the Swedish translation of the General Self-Efficacy scale. Qual Life Res. 2012;21(7):1249-53.

36. Bjelland I, Dahl AA, Haug TT, Neckelmann D. The validity of the Hospital Anxiety and Depression Scale. An updated literature review. Journal of psychosomatic research. 2002;52(2):69-77.
37. Glaesmer H, Rief W, Martin A, Mewes R, Brahler E, Zenger M, et al. Psychometric properties and population-based norms of the Life Orientation Test Revised (LOT-R). British journal of health psychology. 2012;17(2):432-45.
38. Blomstedt Y, Emmelin M, Weinehall L. What about healthy participants? The improvement and deterioration of self-reported health at a 10-year follow-up of the Vasterbotten Intervention Programme. Global health action. 2011;4:5435.
39. Shah LC, West P, Bremmeyr K, Savoy-Moore RT. Health literacy instrument in family medicine: the "newest vital sign" ease of use and correlates. J Am Board Fam Med. 2010;23(2):195-203.
40. Nambi V, Chambless L, He M, Folsom AR, Mosley T, Boerwinkle E, et al. Common carotid artery intima-media thickness is as good as carotid intima-media thickness of all carotid artery segments in improving prediction of coronary heart disease risk in the Atherosclerosis Risk in Communities (ARIC) study. Eur Heart J. 2012;33(2):183-90.
41. Touboul PJ, Hennerici MG, Meairs S, Adams H, Amarenco P, Bornstein N, et al. Mannheim carotid intima-media thickness and plaque consensus (2004-2006-2011). An update on behalf of the advisory board of the 3rd, 4th and 5th watching the risk symposia, at the 13th, 15th and 20th European Stroke Conferences, Mannheim, Germany, 2004, Brussels, Belgium, 2006, and Hamburg, Germany, 2011. Cerebrovasc Dis. 2012;34(4):290-6.
42. Tsimikas S, Miller YI. Oxidative modification of lipoproteins: mechanisms, role in inflammation and potential clinical applications in cardiovascular disease. Curr Pharm Des. 2011;17(1):27-37.
43. Fernvik EC, Ketelhuth DF, Russo M, Gidlund M. The autoantibody repertoire against copper- or macrophage-modified LDL differs in normolipidemics and hypercholesterolemic patients. J Clin Immunol. 2004;24(2):170-6.
44. Mencarelli A, Cipriani S, Renga B, D'Amore C, Palladino G, Distrutti E, et al. FXR activation improves myocardial fatty acid metabolism in a rodent model of obesity-driven cardiotoxicity. Nutr Metab Cardiovasc Dis. 2013;23(2):94-101.
45. Thomsen M, Varbo A, Tybjaerg-Hansen A, Nordestgaard BG. Low nonfasting triglycerides and reduced all-cause mortality: a mendelian randomization study. Clin Chem. 2014;60(5):737-46.
46. Stein JH, Korcarz CE, Hurst RT, Lonn E, Kendall CB, Mohler ER, et al. Use of carotid ultrasound to identify subclinical vascular disease and evaluate cardiovascular disease risk: a consensus statement from the American Society of Echocardiography Carotid Intima-Media Thickness Task Force. Endorsed by the Society for Vascular Medicine. Journal of the American Society of Echocardiography : official publication of the American Society of Echocardiography. 2008;21(2):93-111; quiz 89-90.
47. Vanoli D, Lindqvist P, Wiklund U, Henein M, Naslund U. Fully automated on-screen carotid intima-media thickness measurement: a screening tool for subclinical atherosclerosis. J Clin Ultrasound. 2013;41(6):333-9.
48. Polak JF, Pencina MJ, O'Leary DH, D'Agostino RB. Common carotid artery intima-media thickness progression as a predictor of stroke in multi-ethnic study of atherosclerosis. Stroke. 2011;42(11):3017-21.
49. Lorenz MW, Polak JF, Kavousi M, Mathiesen EB, Volzke H, Tuomainen TP, et al. Carotid intima-media thickness progression to predict cardiovascular events in the general population (the PROG-IMT collaborative project): a meta-analysis of individual participant data. Lancet. 2012;379(9831):2053-62.

50. Bots ML, Evans GW, Riley WA, Grobbee DE. Carotid intima-media thickness measurements in intervention studies: design options, progression rates, and sample size considerations: a point of view. Stroke. 2003;34(12):2985-94.
51. Mathiesen EB, Johnsen SH, Wilsgaard T, Bonaa KH, Lochen ML, Njolstad I. Carotid plaque area and intima-media thickness in prediction of first-ever ischemic stroke: a 10-year follow-up of 6584 men and women: the Tromso Study. Stroke. 2011;42(4):972-8.
52. Graneheim UH, Lundman B. Qualitative content analysis in nursing research: concepts, procedures and measures to achieve trustworthiness. Nurse education today. 2004;24(2):105-12.

Visualization of Asymptomatic Atherosclerotic Disease for Optimum Cardiovascular Prevention: VIPVIZA – a Population-based RCT nested in Routine Care in Sweden

NCT01849575

Amendment to Study Protocol version 4.0

Additional major amendments

A Discrepancy between first date registered on cliniclatrilas.gov and first ultrasound examination in VIPVIZA Page 42

B Data on health and physical testing from the Conscripts registry and data for the baseline drop-out analyses from Västerbotten Intervention Programme regarding eligible non-responders and drop-outs. Page 42

C Follow-up six years after Base line Page 43

Version 1.

26/11/2019

A Discrepancy between first date registered on clinicaltrials.gov and first ultrasound examination in VIPVIZA

The VIPVIZA trial was approved by the Regional Ethical Review Board at Umeå University February 7th 2012, Dnr 2011-445-31M. A pilot study was performed September-October 2012, Study Protocol . page17 section 8.6.

The first date regarding VIPVIZA in clinicaltrials.gov was May 8th 2013, while the first baseline ultrasound examination in the main study was performed April 29th 2013. This delay is completely explained by the administrative issues that occurred during registration of the VIPVIZA record at clinicaltrials.gov. Thus, it is documented in mail conversations that the record holder (Margareta Norberg) at April 29th 2013 filled in the form for registration of VIPVIZA at clinicaltrials.gov, but it was not possible for the record holder to determine how to register members of the Steering Committee and the International Advisory Board. This had nothing to do with study procedures, inclusion of study subjects or the conducted examinations. This issue regarding registration of VIPVIZA's administrative and leadership structure was solved on May 2nd 2013, but on the same day the response message by mail from clinicaltrials.gov was that "PRS Administrator for Umeå University must Approve and Release this record". (Moreover April 30th and May 1st are normally out-of-office-days in Sweden). Furthermore, the central administrator for clinicaltrials.gov for Umeå University had only recently been appointed. This delayed the actual registration date further until May 8th 2013.

We therefore claim that the discrepancy of nine days between the date registered at clinicaltrials.gov and the first day for any baseline examination is entirely due to administrative reasons and should not prohibit publication of manuscripts based on the VIPVIZA trial in journals requiring registration before the first examination.
Through documentation in our encrypted database, which is available only to a database manager, it can be demonstrated that the very few subjects, out of the total of 3532 participants included up until June 2016, who were examined during this 9-day period were informed about the trial according to regulations, and had given their written informed consent before being included, thereby ensuring the safety of participants in advance of any treatment or intervention commencing.

B Data from the Swedish Conscripts registry regarding health information and physical testing and data from the Västerbotten Intervention Programme on eligible non-responders and drop-outs at the baseline examination in VIPVIZA

This was approved by the Regional Ethical Review Board Umeå University at December 27th 2018, Dnr 2018-482-32M

C Follow-up six years after baseline

This amendement to the VIPVIZA Study protocol Version 4.0, regards continued follow-up with data collection and examination six years after inclusion in the study using the same research questions as originally and the supplementary changes that have already been made (See Study protocol version 4.0, Major amendments section 15.3 page 36).

Study progress: The three-year follow-up: data collection was completed on June 16 2016. VIPIVZA has followed the study plan. The evaluation of the primary endpoint was completed published in Lancet (Publication 4). Additional publicatins up to November 30 2019 are listed in the Publications list. The two publications by Vanoli et al concern the preparatory evaluations of the ultrasound examination methodology; these evaluations were performed before the study start and did not use data from any VIPIVZA participants. All other publications are based on data collected within the main trial. To date, several substudies are ongoing. The project includes one completed PhD projectand three on-going PhD projects, and a two-year post-doctoral study period that was finalised in June 2019.

The 6-year follow-up includes the following additions, removals or changes.

1. Added measuement or clinical data
   a. Objective measurement of physical activity with accelerometer
   b. Dental health: Two questions in the questionnaire to participants regarding dental health and retrieval of clinical data on dental health and dental radiological examinations from Dental Care.
2. Changes in the questionnaire to participants regarding psychological and social determinants of health behaviours in particular with respect to cardiovascular disease (CVD). Overall, the questionnaires were reduced in scope.
   a. Deleted: the Three-item Brief Health Literacy Screen (BHLS), Questions on anxiety deleted from HADS (Hospital Anxiety and Depression Scale), Brief Cope on coping strategies, LOT-R on optimistic/pessimistic disposition, the 66 item Food Frequency questionnaire that is used in the Västerbotten Intervention Programme (VIP), BMSQ Beliefs about Medicines Questionnaire,
   b. Additions: International Schedule of Social Interaction (ISSI), the Karasek Demand /Control model questionnaire (ISSI and Karasek D/C were included in the baseline VIP questionnaire), questions regarding reactions to the 3-year ultrasound information based on qualitative interviews with participants, SOM/Hp5i a validated questionnaire about personality. Questions to women about pregnancy complications and menopause and reproductive hormonal therapy.
3. New data from registries
   a. Blood group
   b. Data from VIPVIZA participants' previous VIP-examinations before entry to VIPIVZA 10, 20 or even 30 years earlier. Example: A person who was included in VPVIZA in

relation to participating in VIP at the age of 60 years, could have participated in VIP also at ages 50 years, 40 years and even 30 years.

4. Electronic questionnaire: A possibility to answer the questionnaires elctronically. The Umeå University data collection platform is used to guarantee confidentiality and secure data handling according to GDPR.

Motivation for the extension of the trial with a 6-year follow-up: The three measurement points of ultrasound data on subclinical atherosclerosis provide a much greater statistical certainty in assessing trends, notably the effect of the intervention, as compared to only two measuring points. The problem of mis-classification ”towards the null” decreases. Moreover, the atherosclerotic process is so slow that a long follow-up period is necessasry and also, for CVD prevention measures to be effective with regard to affect hard endpoints, they have to be sustainable over long periods.

Ethical considerations regarding risk vs benefit: As previously during the VIPIVZA trial, there is a risk of potentially strong emotional reactions in connection with obtaing ultrasound results. However, this risk is considered to be small as the entire study population was informed about about their personal ultrasound result at the 3-year follow-up and very large individual changes to the 6-year follow-up cannot be expected. This risk is also balanced by the possibility of feedback regarding implemented prevention efforts, both by the individual and provided by health professionals in terms of pharmacological treatment. As before, subjects with severe ultrasound results will also be followed up by research nurses. If strong emotional reactions nevertheless occur, the study team is prepared to take care of these cases.
There is a risk that some parts of the questionnaires, as well as collection of health data from Dental Care, may be perceived as violating privacy. This is balanced by the fact that all data is handled with complete confidentiality and that only aggregated data will be reported, and also that the results may be the basis for development of personalized CVD prevention methods.
As in previous follow-up, as well as at baseline, sampling of blood to the local Medical Biobank for future analyses of novel biomarkers carries only a minor risk of transient tenderness and bruising.
The study projects do not have any direct benefit from these changes of the project. However, even if the usage of an Accelerometer does not entail any direct risk or benefit to participants, the research group's experience from previous projects is that this measurement is appreciated by participants when they get a brief feedback on their own results.

This amendment was approved by the Swedish Ethical Review Authority at September 24th 2019, Dnr 2019-04691.

Visualization of Asymptomatic Atherosclerotic Disease for Optimum Cardiovascular Prevention: VIPVIZA – a Population-based RCT nested in Routine Care in Sweden

NCT01849575

Statistical Analysis Plan

1-year evaluation

Version 4.0 (2018-05-28)

Table of Contents

1. INTRODUCTION 1

2. OBJECTIVES AND OUTCOME VARIABLES 1

2.1. OBJECTIVES 1
2.1.1. SPECIFIC OBJECTIVES 1
2.2. OUTCOME VARIABLES 1
2.2.1. PRIMARY OUTCOME VARIABLES 1
2.2.2. SECONDARY OUTCOME VARIABLES 2
2.2.3. VARIABLES 2
2.3. INDEPENDENT AND OTHER COVARIATES 4

3. STUDY DESIGN 4

3.1. DESIGN 4
3.2. POPULATION 4
3.2.1. INCLUSION CRITERIA 4
3.2.2. EXCLUSION CRITERIA 5
3.3. INTERVENTIONS FEL! BOKMÄRKET ÄR INTE DEFINIERAT.

4. DEFINITIONS OF ANALYSIS POPULATIONS 5

5. DESCRIPTIONS OF STATISTICAL ANALYSIS 6

5.1. STUDY CONDUCT AND SUBJECT DISPOSITION 6
5.2. BASELINE CHARACTERISTICS AND TREATMENT GROUP COMPARABILITY 6
5.3. PRIMARY EFFICACY ANALYSES 6
5.3.1. PRIMARY ANALYSIS 6
5.3.2. SENSITIVITY ANALYSES 6
5.3.3. EFFECTS IN GROUPS DEFINED BY THEIR RISK SCREENING 7
5.3.4. CHECKING OF ASSUMPTIONS 7
5.4. SECONDARY OUTCOME ANALYSES 7
5.4.1. SUB-GROUP ANALYSES 7
FEL! BOKMÄRKET ÄR INTE DEFINIERAT.
5.5. DATA HANDLING AND ANALYTICAL STEPS 8
5.6. IMPUTATION OF MISSING DATA 9
5.7. SAFETY ANALYSIS FEL! BOKMÄRKET ÄR INTE DEFINIERAT.
5.8. OTHER PLANNED ANALYSES 10

6. DESCRIPTIONS OF SAMPLE SIZE FEL! BOKMÄRKET ÄR INTE DEFINIERAT.

7. ANALYSIS DATABASE DEFINITIONS 12

1. Introduction

The purpose of this Statistical Analysis Plan (SAP) is to describe the pre-planned statistical analysis steps and data presentation for the clinical study report of the VIPVIZA trial (ClinicalTrials.gov Identifier: NCT01849575).

First patient in (FPI) was April 2013, and the last pre-FPI protocol version was submitted to ClinicalTrials.gov on 2013-05-08. Subsequent amendments in ClinicalTrials.gov were on 2017-09-25. The estimated primary complete data is June 2019. The estimated study completion date is December 2026.

2. Objectives and outcome variables

2.1. Objectives

This population-based randomized controlled trial (RCT) aims at optimizing cardiovascular disease (CVD) prevention through ensuring accurate identification of individuals at high risk of CVD through carotid ultrasonography examination, promoting accurate perception of the risk through communication of ultrasonography results, and enhancing better compliance to preventive treatments with the ultimate goal to reduce premature CVD morbidity and mortality.

The objective of this SAP is to describe evaluation the effects of the VIPVIZA intervention at one year of follow up. In this section, we describe the primary and secondary objectives and outcome variables for the 1-year evaluation.

2.1.1. Specific objectives

In this study, we test the hypothesis that adding risk communication using direct visualisation of asymptomatic atherosclerotic disease, assessed through carotid ultrasound, is superior in reducing a combined index of CVD risk factors at 1-year follow-up compared to the routine clinical guidelines followed at the primary health care setting. The combined measures of CVD risk factors are the Framingham risk score (FRS) and the SCORE.

2.2. Outcome variables

2.2.1. Primary outcome variables

The primary outcomes include:
- Framingham Risk Score
- SCORE risk

To achieve the overall aim, we will evaluate the SCORE risk and the Framingham scores between the intervention groups at baseline and 1-year.

We will generate the SCORE risk using gender-specific regression weights to estimate the risk for cardiovascular risk of men and women. The SCORE includes assessment on the levels of total cholesterol, systolic blood pressure, smoking and age.

We will also generate Framingham score using gender-specific algorithm to estimate the 10-year cardiovascular risk of men and women. The Framingham score includes assessment on the levels of total cholesterol, LDL-cholesterol, systolic blood pressure, treatment for high blood pressure, diabetes, smoking and age.

2.2.2. Secondary outcome variables

The secondary outcomes include:
- Changes in the CVD risk factors (blood pressure, serum cholesterol, LDL, HDL, triglycerides, fasting glucose, HbA1c) between the baseline and 1-year follow-up.
- Changes in the lifestyle behaviours (physical activity, tobacco use, alcohol use, eating habits) between the baseline and 1-year follow-up.

2.2.3. Variables

To test the hypothesis, we will use arrays of lifestyle behaviours, physical measurements and results from blood samples assessed in the baseline and 1-year follow-up.

Self-reported lifestyle behaviours
- Smoking behaviour
- Snus consumptions
- Use of alcohol, assessed with the AUDIT questionnaire
- Physical activity level and sedentary behaviour
- Dietary habits, indicated by daily consumption of fruits, roots, legymes and vegetables

Physical measurements
- Height measurement (in cm)
- Weight measurement (in kg)
- Waist circumference (in cm)
- Systolic blood pressure (in mmHg)
- Diastolic blood pressure (in mmHg)

Blood examinations
- Total serum cholesterol (in mmol/l)
- Serum triglyceride (in mmol/l)
- LDL-cholesterol (in mmol/l)
- HDL-cholesterol (in mmol/l)
- Fasting blood glucose (in mmol/l)
- 2-hour post-prandial blood glucose (in mmol/l)

Prescriptions and purchases of medications for the treatment of:

- hypertension and/or dyslipidemia and/or diabetes

Ultrasound results

- CIMT (carotic intima-media wall thickness)
- Presence of plaque (in categories: yes or no)
- Quantitative and qualitative plaque parameters
- Presence and degree of significant stenosis

2.3. Independent and other covariates

Treatment groups
- Group: intervention or control group

Sociodemographic variables
- Birth year
- Sex
- Age groups
- Highest education level

Other variables
- History/being diagnosed of diabetes among the participant
- Family history of heart infarction before 60 years old among parents and siblings
- Family history of diabetes among parents and siblings
- Portion of potato/rice, meat/fish, and vegetable consumptions
- Date of baseline or follow-up examination

3. Study Design

3.1. Design

- A pragmatic randomised clinical trial within the entire primary health care in Västerbotten County.
- Participants were randomized to two equal groups (intervention and control group) using a randomisation list generated prior to the study using simulation from a uniform probability distribution.
- Both groups will be managed throughout the study according to clinical guidelines on CVD risk factor control within primary health care.

3.2. Population

3.2.1. Inclusion criteria

Inclusion criteria were based on 1/ the aim of targeting subjects at intermediate risk of CVD, and 2/established clinical criteria (smoking, diabetes, hypertension, abdominal obesity) or evaluation of levels and distributions of clinical CVD risk markers (S-LDL) in the 2011 VIP population. In this population, the cut-off for the 4th quartile among 50 years old men and women was 4.4 mmol/L and 4.0 mmol/L, respectively, and 9% and 12%, respectively, had S-LDL concentrations ≤2.5 mmol/L, and 81% and 88%, respectively, S-LDL ≤4.5 mmol/L.

1. age=40 and a history of CVD at age <60 years among first-degree relative(s)
2. age=50 years and at least one of the following: a history of CVD at age <60 years among first-degree relative(s), smoking, diabetes, hypertension, S-LDL-cholesterol ≥4.5 mmol/L, abdominal obesity defined by waist >88cm for women and >102 cm for men
3. age=60 years

3.2.2. Exclusion criteria

A significant stenosis as defined by >50% luminal narrowing of the investigated carotid arteries according to the first vascular ultrasound. These individuals are, irrespective of their randomisation status, informed with a phone call about results. They are therefore not included in the study population and hence not invited to the follow-up examination in the trial. We also exclude individuals who violate the study protocol during the trial as well as those who participate in another clinical trial after the baseline examination.

3.3. Treatment

- **Intervention group:**
    - Information about carotid ultrasound results were given to the participant and his/her primary care physician including graphic presentations of atherosclerosis highlighted in colour against normal vascular age patterns as a gauge going from a green sector over yellow and orange to a red sector that illustrate the percentiles, 1-25, 26–50, 51–75 and 76–100, respectively. Plaque formation was shown as a traffic light for each side, with a green circle for not detected and a red circle for detected plaque. A stylized picture of the participant's own ultrasound image showed vascular age as a coloured line and plaques as a red mark. Brief written information about atherosclerosis as a dynamic process modifiable by healthy lifestyle and pharmacological treatment was also given.
    - After 2-4 weeks, participants received a follow-up phone call by a research nurse in order to reassure and give additional information if needed.
    - The same graphic information was repeated after 6 months.
    - Treatment according to clinical guidelines.

- **Control group:**
- No information about the carotid ultrasound was given at baseline.
- Treatment according to clinical guidelines.

4. Definitions of Analysis Populations

- Intention to Treat analysis (ITT). The full analysis set will be used for all primary presentation of data and analysis. Individuals enrolled in the routinely implemented VIP screening among the population was invited to the study. Those giving their consent were randomized to join either the intervention group or the control group with equal (50%) probability. A randomization list for the treatment group was created in the R-program for statistical computing.

5. Descriptions of Statistical Analysis

5.1. Study conduct and subject disposition

- Descriptive statistics of inclusion and follow-up will be given by randomised treatment for ITT. First and last inclusion time will be tabulated, and inclusion plotted as a cumulative plot.
- Patients with non-completion time to withdrawal or loss to follow-up, and time to last follow-up date, will be summarized in tables summarised using min, median and max, will be tabulated as number and percentage, place and individual characteristics.

5.2. Baseline characteristics and treatment group comparability

- Categorical variables will be described as number and percentage by randomized groups.
- Numerical variables will be presented as mean, median, standard deviation and range.
- The frequency of missing data will be presented in a separate column for all the variables.
- No statistical tests (p-value) will be estimated in the baseline table.

5.3. Primary efficacy analyses

5.3.1. Primary analysis

The differences in primary outcomes (SCORE risk and Framingham Risk Score) at one year between the treatment groups will be measured and analysed using t-tests. As the group sizes are large (more than thousand), it is valid to assume normal distribution of the error around the mean value estimates in both the intervention and the control group. Complementary to the absolute difference in the Score, Cohen´s effect size will be derived for the difference in mean outcome values divided by a pooled standard deviation of the Score between the 2 groups. A two-sided p-value <0.05 will be regarded as statistically significant.

5.3.2. Sensitivity analyses

Adjusted analysis will be conducted using linear regression with covariates. This is because many of the covariates are related to the prognosis of outcome (including sex and age) are related to the prognosis of outcomes, and in such situations, adjustments can yield coefficients slightly further from 0. We will use the continuous form of the primary outcomes (SCORE risk and Framingham Risk Score) as outcome in the regression analysis. We will assess the effects of the intervention independently for each of the outcome measure.
We will also conduct unadjusted linear regression as sensitivity analysis. Comparing the unadjusted and adjusted results provides assurance of the robustness of the results, especially when one of the results achieves only borderline significance.

We will conduct sub-groups analyses to compare the effects of the intervention among different population groups. More details about the sub-group analyses are presented in Section 5.4.1.

5.3.3. Effects in groups defined by their vascular ultrasound result

- Pairwise t-test will be used to assess the intervention effect in groups stratified by the vascular ultrasound result at baseline. The vascular ultrasound measurements includes information of the plaque presence and the age and sex standardized vascular age of the subject.
  For this test, the vascular age groups will be created according to the age standardized quartiles: green (young vascular compared to actual age), yellow (intermediate towards young), orange (intermediate towards red), red (old vascular compared to actual age). The t-test analysis will compare red to red, orange to orange, yellow to yellow, green to green of the intervention and control group, respectively. The t-test will be adjusted for multiple testing using the Bonferroni method.

5.3.4. Checking of assumptions

Only assumption is normal distribution which can be assumed for mean values estimates if groups are larger than 30. Since our groups is much larger than 30 we see not need to assess this assumption. We will calculate the t-test using the group specific standard deviations, to relax the assumption of equal variances.

5.4. Secondary outcome analyses

5.4.1. Sub-group analyses

In the analysis, we will compare the primary and secondary outcome variables' mean value between the intervention and the control in pre-specified sub-groups.

The sub-groups are:
- Abdominal obesity based on waist circumference measurement to compare the non-obese and obese groups, using the cut-off of waist circumference ≥102 cm for men and ≥88 cm for women according to the WHO definition.
- General obesity based on the body mass index measurement to compare the underweight, normal weight, overweight and obese groups, using the cut-off points of 18.5, 25, and 30, respectively.
- Physical activity level to compare respondents with low and high-level of physical activity
- Sex to compare men and women
- Age group to compare respondents at age 40, 50 and 60 years old
- Education level to compare respondents with low, medium and high education level as defined earlier.

- Since participants are recruited into the study during the three years from mid-2013 to mid-2016, any change in physicians' prescribing behaviour over the course of the study will be evaluated by comparing prescriptions issued for lipid-lowering and anti-hypertensive medications during the first year after the baseline ultrasound examination as well as primary outcome among participants recruited during the first, second and third years

5.5. Data handling and analytical steps

The data is entered using Microsoft SQL and is stored in common database file formats including SPSS and Stata. We will follow the following steps in handling the data to ensure its validity and make an analytical dataset for the analysis of this paper.

Step 1: We will conduct **quality check** on the baseline and 1-year follow-up data. Information from baseline and 1-year follow-up will be merged using the unique study number allocated for each individual (the VIPVIZAID). Individuals who did not participate in the 1-year follow-up will be noted and reasons for not participating will be identified and listed in the dataset. The completeness of all variables will be checked through, and if needed, the research nurse will double check any missing values in the dataset against the individual's responses in the paper questionnaire.

Step 2: We will check the dataset for **outliers and illogical values**. For categorical variables, responses outside the possible response categories will be coded as missing. For continuous variables, values outside the plausible ranges will be coded as missing also.

For biological markers, we will use the following cut-off points to define outliers. Any values outside these ranges will be coded as missing.

Variables	Plausible values
Fasting blood glucose (mmol/L)	2.0 – 25.0
Serum cholesterol (mmol/L)	2.0 – 18.0
LDL cholesterol (mmol/L)	1.2 – 15.0
HDL cholesterol (mmol/L)	0.4 – 6.0
Serum triglycerides (mmol/L)	0.4 – 20.0
Systolic blood pressure (mmHg)	80 – 240
Diastolic blood pressue (mmHg)	55* – 150
	*unless if systolic blood pressure below 90
Pulse pressure (mmHg)	15-100
Height (cm)	110 – 210
Change in height from baseline to 1-yr exam (cm)	>4
Weight (kg)	40 – 200
Waist (cm)	50 - 170
Change waist from baseline to 1-yr exam > 5cm	Change weight >15 kg
Change weight from baseline to 1-yr exam >5 kg	Change waist >15 cm

Step 3: After all the data have been cleaned, we will conduct **imputation of missing data** (see section 5.6). To the best possible, we will impute the original variables, not the derived variables. For example, instead of imputing the variable body mass index, we will impute the variable weight and height which is used to calculate body mass index. See the next section for more detailed descriptions on missing data.

Step 4: After the data have been imputed, we will generate **derived variables** based on the imputed data. For example, body mass index will be calculated from weight and height in the imputed data.

5.6. Imputation of Missing Data

Assessment of missing data will be conducted using the missing data modules in the Stata Statistical Programme. The command *misschk*, *misstable* will be used to assess the patterns of missing data among all the variables included in the analysis. To ensure reproducibility of data analysis conducted in VIPVIZA, we plan to create one imputed dataset based on assessment of all variables in the dataset and subsequently use this imputed dataset for analysis of future papers. Information about number of study participants with missing data for each variable will be presented in the description table showing the baseline characteristics of the study participants in the intervention and control groups.

We will conduct multiple imputation based on Rubin (1987) and Schafer (1997) methods using the *mi* estimate command sets in Stata. In brief, multiple imputation involves three steps: (i) imputation step: selection of imputation model to generate 10-20 imputed complete datasets to capture the uncertainty of the imputation model; (ii) estimation step (completed-data analysis): the planned analyses are conducted separately on each imputed dataset; and (3) pooling step: the results obtained from the series of completed-data analyses are combined into a single multiple-imputation result.

The command ***mi estimate*** estimates model parameters from multiply imputed data and adjusts coefficients and standard errors for the variability between imputations (***the estimation step***). It runs the specified estimation command on each of the M imputed datasets to obtain the M completed-data estimates of coefficients and their variance–covariance matrix of the estimators (VCEs). It then computes MI estimates of coefficients and standard errors by applying combination rules to the M completed-data estimates (***the pooling step***). Methods and formulas for computation details are described in the Stata's Multiple Imputations' guide, available at https://www.stata.com/manuals13/mimiestimate.pdf

If data is **missing not at random**, a thorough assessment between study respondents with and without missing data on specific variables will be conducted based on the available information such as sociodemographic variables. The intention is to identify if this non-ignorable missing data might introduce any systematic bias that might influence the results of the estimation. We will employ selection models and/or pattern mixture models as multiple imputation strategies. Under the condition of MNAR, the joint density of VIPVIZA's participant responses is not the same for participants with full and partially observed data. We will perform pattern mixture models by modelling the observed data, then model the

missing data as a modification of the observed data model. We will explicitly model the missing data distribution by first identifying different patterns of missing data and then including parameters in the outcomes model that capture this effect (Paddock et al. 2006).

We will also conduct sensitivity analysis by comparing the results with complete case analysis vs. results with multiple imputation with different sets of imputation.

Reference for multiple imputations:
Stata Multiple-Imputation Reference Manual Release 13, StataCorp LP, Texas: https://www.stata.com/manuals13/mi.pdf).

Paddock SM, Edelen MO, Wenzel SL, Ebener PA, Mandell W. 2006. Pattern-Mixture Models for Addressing Nonignorable Nonresponse in Longitudinal Substance Abuse Treatment Studies. RAND Health.

5.7. Safety Analysis

The intervention in VIPVIZA is pictorial information about ultrasound results on the individual's actual atherosclerosis. This is considered to be a low-intensity intervention in comparison to interventions with pharmacological drugs or surgical procedures. The ultrasound examination cannot cause any harm, physical discomfort or risk. As with all screening targeting a healthy population, it is a dilemma that asymptomatic individuals may be informed of silent disease, in this case ongoing atherosclerotic process with increased risk of future CVD. This can be perceived more serious than just an increased level of risk markers, and result in anxiety.

In order to avoid unjustified concerns, all persons in the intervention group receive a telephone call with a research nurse and, if necessary, a doctor in charge, for in-depth and balanced information about ultrasound results. This conversation is held according to the methodology of motivational interviewing and also aims at increased awareness of the possibility of reducing the individual risk by means of own preventive measures. This is expected to alleviate anxiety and increase motivation to follow the recommendations for preventive treatment.

According to the study hypotheses, this is expected to benefit the individual due to risk reduction. A healthier lifestyle is also expected to benefit the individual through increased well-being and quality of life. Similarly, subjects without ongoing atherosclerotic disease will be able to avoid unjustified concerns and informed to continue a healthy lifestyle.

All individuals with severe carotid stenosis will be excluded from the study and are referred directly to the Stroke Centre for assessment and treatment. This may potentially be life saving for these people, and may, to some extent, balance the fact that no information is given until after 3 years to half the group even if small/moderate changes are detected.

5.8. Other planned analyses

- In **medium-term**, adding risk communication using visualisation of carotid ultrasound results is more effective in reducing **hospitalisation due to stroke, myocardial**

infarctions and revascularisations (at 5-year and 10-year) compared to routine CVD risk assessment and control within the primary health care setting. – Not for 1-year evaluation.

- In **long-term**, adding risk communication using visualisation of carotid ultrasound results is more effective in reducing **overall mortality and cause-specific mortality due to myocardial infarctions and stroke** (at 5-year and 10-year) compared to routine CVD risk assessment and control within the primary health care setting. – Not for 1-year evaluation.

6. Descriptions of Sample Size

Calculations based on data on conventional risk factors derived from VIP 2011, revealed that 3500 included study participants with a drop-out rate of 15 % during the study would be sufficient to assure a probability of 80% to detect a true difference between groups at a significance level of 5%. The limiting factor (demanding the largest group size to show a hypothesized effect) was CIMT.

Variabel	Significance level	Power	SD	Relevant change Population level	Sample size per group	Minimum detectable change if n=1500/group
SBP (mmHg)	0.05	0.8	16	2	1500	1.7
Serum Cholesterol (mmol/L)	0.05	0.8	0.5	0.5	1500	0.05
LDL (mmol/L)	0.05	0.8	0.5	0.5	1500	0.05
CIMT (mm)	0.05	0.8	0.2	0.02	1500	0.02
SCORE	0.05	0.8	1.40	0.5	1500	0.143
Framingham Risk Score	0.05	0.8	6.60	1	1500	0.7

7. Analysis Database Definitions

Variable	Description
age_0	Age in days at VIP baseline (*Rec)
age_1	Age in days at 1 year examination (*Rec)
alder_0	Age group (*Rec)
byear_0	Year of birth
in_kon	Gender (*Rec)
in_part0	Eligible participant and participation in main study (baseline) (*Rec)
in_part1	Eligible participant and participation in 1 year follow-up (*Rec)
in_randg	Randomization group (*Rec)
in_status	Participation status
educa_0	Education status (*Rec)
health_0	Perceived health during last year
g1d_0	Travel to and from work - Winter (*Rec)
g1d_1	Travel to and from work - Winter (*Rec)
g1km_0	How many kilometers do you have to travel to commute? (One way) (*Rec)
g1km_1	How many kilometers do you have to travel to commute? (One way) (*Rec)
g3a_0	What recreational activities do you participate in - Walks (*Rec)
g3a_1	What recreational activities do you participate in - Walks
g3b_0	What recreational activities do you participate in - Bicycling (*Rec)
g3b_1	What recreational activities do you participate in - Bicycling
g6_0	How often have you worked out or exercised in your training-clothes during the last three months (*Rec)
g6_1	How often have you worked out or exercised in your training-clothes during the last three months (*Rec)
g9_0	To what extent have you been physically active during leisure time during the past 12 months? (*Rec)
g9_1	To what extent have you been physically active during leisure time during the past 12 months? (*Rec)
g10_0	During an ordinary week, how much time do you spend on moderately strenuous activities (*Rec)
g10_1	During an ordinary week, how much time do you spend on moderately strenuous activities (*Rec)
h1antal_0	Number of cigarettes / day
h1antal_1	Number of cigarettes / day
h1a_0	Do you presently smoke? No, I never have smoked
h1a_1	Do you presently smoke? No, I never have smoked
h1b_0	Do you presently smoke? Yes, I smoke cigarettes
h1b_1	Do you presently smoke? Yes, I smoke cigarettes
h1c_0	Do you presently smoke? Yes, I smoke cigars
h1c_1	Do you presently smoke? Yes, I smoke cigars

h1d_0	Do you presently smoke? Yes, I smoke a pipe
h1d_1	Do you presently smoke? Yes, I smoke a pipe
h1e_0	Do you presently smoke? Yes, I smoke occasionally (Not daily)
h1e_1	Do you presently smoke? Yes, I smoke occasionally (Not daily)
h1f_0	Do you presently smoke? Not now, but I used to smoke daily
h1f_1	Do you presently smoke? Not now, but I used to smoke daily
h1g_0	Do you presently smoke? Not now, but I used to smoke occasionally
h1g_1	Do you presently smoke? Not now, but I used to smoke occasionally
h4_0	Have you ever used snuff? (*Rec)
h4_1	Have you ever used snuff? (*Rec)
j01_0	How often do you have a drink containing alcohol?
j01_1	How often do you have a drink containing alcohol?
j02_0	How many drinks containing alcohol do you have on a typical day when you are drinking?
j02_1	How many drinks containing alcohol do you have on a typical day when you are drinking?
j03_0	How often do you have six or more drinks on one occasion?
j03_1	How often do you have six or more drinks on one occasion?
j04_0	How often during the last year have you found that you were not able to stop drinking once you had started?
j04_1	How often during the last year have you found that you were not able to stop drinking once you had started?
j05_0	How often during the last year have you failed to do what was normally expected of you because of drinking?
j05_1	How often during the last year have you failed to do what was normally expected of you because of drinking?
j06_0	How often during the last year have you needed a first drink in the morning to get yourself going after a heavy drinking session
j06_1	How often during the last year have you needed a first drink in the morning to get yourself going after a heavy drinking session
j07_0	How often during the last year have you had a feeling of guilt or remorse after drinking?
j07_1	How often during the last year have you had a feeling of guilt or remorse after drinking?
j08_0	How often during the last year have you been unable to remember what happened the night before because of drinking?
j08_1	How often during the last year have you been unable to remember what happened the night before because of drinking?
j09_0	Have you or someone else been injured because of your drinking?
j09_1	Have you or someone else been injured because of your drinking?
j10_0	Has a relative, friend, doctor or other health care worker been concerned about your drinking or suggested to cut down?
j10_1	Has a relative, friend, doctor or other health care worker been concerned about your drinking or suggested to cut down?
langd_0	Body height cm (*Rec)
langd_1	Body height cm (*Rec)

vikt_0	Body weight kg (*Rec)
vikt_1	Body weight kg (*Rec)
midja_0	Waist circumference cm (*Rec)
midja_1	Waist circumference cm (*Rec)
sbt_0	Systolic blood pressure mmHg (*Rec)
sbt_1	Systolic blood pressure mmHg (*Rec)
dbt_0	Diastolic blood pressure mmHg (*Rec)
dbt_1	Diastolic blood pressure mmHg (*Rec)
skol_0	Serum cholesterol mmol/l (*Rec)
skol_1	Serum cholesterol mmol/l (*Rec)
stg_0	Serum triglycerides mmol/l (*Rec)
stg_1	Serum triglycerides mmol/l (*Rec)
ldl_0	LDL-cholesterol mmol/l (*Rec)
ldl_1	LDL-cholesterol mmol/l (*Rec)
hdl_0	HDL-cholesterol mmol/l (*Rec)
hdl_1	HDL-cholesterol mmol/l (*Rec)
blods0_0	Blood sugar fasting mmol/l (*Rec)
blods0_1	Blood sugar fasting mmol/l (*Rec)
c10_1_0	Medication in the last 2 weeks - Hypertension (*Rec)
c10_1_1	Medication in the last 2 weeks - Hypertension (*Rec)
c10_5_0	Medication in the last 2 weeks - Lipid lowering drug (*Rec)
c10_5_1	Medication in the last 2 weeks - Lipid lowering drug (*Rec)
c10_8_1	Medication in the last 2 weeks - Diabetes medication (*Rec)
c2_0	Did any of your parents or siblings suffer a heart attack/myocardial infarction or stroke before age 60 years?
c3_0	Does any of your parents or siblings suffer from diabetes?
c6_0	Do you suffer from diabetes?
c7a_0	If you have answered Yes on question C6, what is your treatment? Diet and exercise
c7b_0	If you have answered Yes on question C6, what is your treatment? Tablets
c7c_0	If you have answered Yes on question C6, what is your treatment? Insulin
c7d_0	If you have answered Yes on question C6, what is your treatment? None of the above
smoking_0	Smoking
smoking_1	Smoking 1 year (*Rec)
snus_0	Use of snus
phyact_0	Physical activity (*Rec)
phyact_1	Physical activity 1 year (*Rec)
fruitandveg_0	Fruit and vegetable consumption (*Rec)
lrmx_0	IMT CCA max mean value independent of side and angle
lrplack_0	Plack left/right combined
imt_color_0	IMT color code for patient information
usage_0	Age in days at ultrasound baseline

usdat_0	Ultrasound date (*Rec)
provdat_0	Sample date on optical questionnaire
provdat_1	Sample date on optical questionnaire (*Rec)

Visualization of Asymptomatic Atherosclerotic Disease for Optimum Cardiovascular Prevention: VIPVIZA – a Population-based RCT nested in Routine Care in Sweden

NCT01849575

Statistical Analysis Plan

1-year evaluation

Version 4.0 (2018-05-28)

Table of Contents

1. INTRODUCTION 1

2. OBJECTIVES AND OUTCOME VARIABLES 1

2.1. OBJECTIVES 1
2.1.1. SPECIFIC OBJECTIVES 1
2.2. OUTCOME VARIABLES 1
2.2.1. PRIMARY OUTCOME VARIABLES 1
2.2.2. SECONDARY OUTCOME VARIABLES 2
2.2.3. VARIABLES 2
2.3. INDEPENDENT AND OTHER COVARIATES 4

3. STUDY DESIGN 4

3.1. DESIGN 4
3.2. POPULATION 4
3.2.1. INCLUSION CRITERIA 4
3.2.2. EXCLUSION CRITERIA 5
3.3. INTERVENTIONS FEL! BOKMÄRKET ÄR INTE DEFINIERAT.

4. DEFINITIONS OF ANALYSIS POPULATIONS 5

5. DESCRIPTIONS OF STATISTICAL ANALYSIS 6

5.1. STUDY CONDUCT AND SUBJECT DISPOSITION 6
5.2. BASELINE CHARACTERISTICS AND TREATMENT GROUP COMPARABILITY 6
5.3. PRIMARY EFFICACY ANALYSES 6
5.3.1. PRIMARY ANALYSIS 6
5.3.2. SENSITIVITY ANALYSES 6
5.3.3. EFFECTS IN GROUPS DEFINED BY THEIR RISK SCREENING 7
5.3.4. CHECKING OF ASSUMPTIONS 7
5.4. SECONDARY OUTCOME ANALYSES 7
5.4.1. SUB-GROUP ANALYSES 7
FEL! BOKMÄRKET ÄR INTE DEFINIERAT.
5.5. DATA HANDLING AND ANALYTICAL STEPS 8
5.6. IMPUTATION OF MISSING DATA 9
5.7. SAFETY ANALYSIS FEL! BOKMÄRKET ÄR INTE DEFINIERAT.
5.8. OTHER PLANNED ANALYSES 10

6. DESCRIPTIONS OF SAMPLE SIZE FEL! BOKMÄRKET ÄR INTE DEFINIERAT.

7. ANALYSIS DATABASE DEFINITIONS 12

1. Introduction

The purpose of this Statistical Analysis Plan (SAP) is to describe the pre-planned statistical analysis steps and data presentation for the clinical study report of the VIPVIZA trial (ClinicalTrials.gov Identifier: NCT01849575).

First patient in (FPI) was April 2013, and the last pre-FPI protocol version was submitted to ClinicalTrials.gov on 2013-05-08. Subsequent amendments in ClinicalTrials.gov were on 2017-09-25. The estimated primary complete data is June 2019. The estimated study completion date is December 2026.

2. Objectives and outcome variables

2.1. Objectives

This population-based randomized controlled trial (RCT) aims at optimizing cardiovascular disease (CVD) prevention through ensuring accurate identification of individuals at high risk of CVD through carotid ultrasonography examination, promoting accurate perception of the risk through communication of ultrasonography results, and enhancing better compliance to preventive treatments with the ultimate goal to reduce premature CVD morbidity and mortality.

The objective of this SAP is to describe evaluation the effects of the VIPVIZA intervention at one year of follow up. In this section, we describe the primary and secondary objectives and outcome variables for the 1-year evaluation.

2.1.1. Specific objectives

In this study, we test the hypothesis that adding risk communication using direct visualisation of asymptomatic atherosclerotic disease, assessed through carotid ultrasound, is superior in reducing a combined index of CVD risk factors at 1-year follow-up compared to the routine clinical guidelines followed at the primary health care setting. The combined measures of CVD risk factors are the Framingham risk score (FRS) and the SCORE.

2.2. Outcome variables

2.2.1. Primary outcome variables

The primary outcomes include:
- Framingham Risk Score
- SCORE risk

To achieve the overall aim, we will evaluate the SCORE risk and the Framingham scores between the intervention groups at baseline and 1-year.

We will generate the SCORE risk using gender-specific regression weights to estimate the risk for cardiovascular risk of men and women. The SCORE includes assessment on the levels of total cholesterol, systolic blood pressure, smoking and age.

We will also generate Framingham score using gender-specific algorithm to estimate the 10-year cardiovascular risk of men and women. The Framingham score includes assessment on the levels of total cholesterol, LDL-cholesterol, systolic blood pressure, treatment for high blood pressure, diabetes, smoking and age.

2.2.2. Secondary outcome variables

The secondary outcomes include:
- Changes in the CVD risk factors (blood pressure, serum cholesterol, LDL, HDL, triglycerides, fasting glucose, HbA1c) between the baseline and 1-year follow-up.
- Changes in the lifestyle behaviours (physical activity, tobacco use, alcohol use, eating habits) between the baseline and 1-year follow-up.

2.2.3. Variables

To test the hypothesis, we will use arrays of lifestyle behaviours, physical measurements and results from blood samples assessed in the baseline and 1-year follow-up.

Self-reported lifestyle behaviours
- Smoking behaviour
- Snus consumptions
- Use of alcohol, assessed with the AUDIT questionnaire
- Physical activity level and sedentary behaviour
- Dietary habits, indicated by daily consumption of fruits, roots, legymes and vegetables

Physical measurements
- Height measurement (in cm)
- Weight measurement (in kg)
- Waist circumference (in cm)
- Systolic blood pressure (in mmHg)
- Diastolic blood pressure (in mmHg)

Blood examinations
- Total serum cholesterol (in mmol/l)
- Serum triglyceride (in mmol/l)
- LDL-cholesterol (in mmol/l)
- HDL-cholesterol (in mmol/l)
- Fasting blood glucose (in mmol/l)
- 2-hour post-prandial blood glucose (in mmol/l)

Prescriptions and purchases of medications for the treatment of:
- hypertension and/or dyslipidemia and/or diabetes

Ultrasound results
- CIMT (carotic intima-media wall thickness)
- Presence of plaque (in categories: yes or no)
- Quantitative and qualitative plaque parameters
- Presence and degree of significant stenosis

2.3. Independent and other covariates

Treatment groups

- Group: intervention or control group

Sociodemographic variables

- Birth year
- Sex
- Age groups
- Highest education level

Other variables

- History/being diagnosed of diabetes among the participant
- Family history of heart infarction before 60 years old among parents and siblings
- Family history of diabetes among parents and siblings
- Portion of potato/rice, meat/fish, and vegetable consumptions
- Date of baseline or follow-up examination

3. Study Design

3.1. Design

- A pragmatic randomised clinical trial within the entire primary health care in Västerbotten County.
- Participants were randomized to two equal groups (intervention and control group) using a randomisation list generated prior to the study using simulation from a uniform probability distribution.
- Both groups will be managed throughout the study according to clinical guidelines on CVD risk factor control within primary health care.

3.2. Population

3.2.1. Inclusion criteria

Inclusion criteria were based on 1/ the aim of targeting subjects at intermediate risk of CVD, and 2/established clinical criteria (smoking, diabetes, hypertension, abdominal obesity) or evaluation of levels and distributions of clinical CVD risk markers (S-LDL) in the 2011 VIP population. In this population, the cut-off for the 4th quartile among 50 years old men and women was 4.4 mmol/L and 4.0 mmol/L, respectively, and 9% and 12%, respectively, had S-LDL concentrations ≤2.5 mmol/L, and 81% and 88%, respectively, S-LDL ≤4.5 mmol/L.

1. age=40 and a history of CVD at age <60 years among first-degree relative(s)
2. age=50 years and at least one of the following: a history of CVD at age <60 years among first-degree relative(s), smoking, diabetes, hypertension, S-LDL-cholesterol ≥4.5 mmol/L, abdominal obesity defined by waist >88cm for women and >102 cm for men
3. age=60 years

3.2.2. Exclusion criteria

A significant stenosis as defined by >50% luminal narrowing of the investigated carotid arteries according to the first vascular ultrasound. These individuals are, irrespective of their randomisation status, informed with a phone call about results. They are therefore not included in the study population and hence not invited to the follow-up examination in the trial. We also exclude individuals who violate the study protocol during the trial as well as those who participate in another clinical trial after the baseline examination.

3.3. Treatment

- **Intervention group:**
  - Information about carotid ultrasound results were given to the participant and his/her primary care physician including graphic presentations of atherosclerosis highlighted in colour against normal vascular age patterns as a gauge going from a green sector over yellow and orange to a red sector that illustrate the percentiles, 1-25, 26–50, 51–75 and 76–100, respectively. Plaque formation was shown as a traffic light for each side, with a green circle for not detected and a red circle for detected plaque. A stylized picture of the participant’s own ultrasound image showed vascular age as a coloured line and plaques as a red mark. Brief written information about atherosclerosis as a dynamic process modifiable by healthy lifestyle and pharmacological treatment was also given.
  - After 2-4 weeks, participants received a follow-up phone call by a research nurse in order to reassure and give additional information if needed.
  - The same graphic information was repeated after 6 months.
  - Treatment according to clinical guidelines.

- **Control group:**
- No information about the carotid ultrasound was given at baseline.
- Treatment according to clinical guidelines.

4. Definitions of Analysis Populations

- Intention to Treat analysis (ITT). The full analysis set will be used for all primary presentation of data and analysis. Individuals enrolled in the routinely implemented VIP screening among the population was invited to the study. Those giving their consent were randomized to join either the intervention group or the control group with equal (50%) probability. A randomization list for the treatment group was created in the R-program for statistical computing.

5. Descriptions of Statistical Analysis

5.1. Study conduct and subject disposition

- Descriptive statistics of inclusion and follow-up will be given by randomised treatment for ITT. First and last inclusion time will be tabulated, and inclusion plotted as a cumulative plot.
- Patients with non-completion time to withdrawal or loss to follow-up, and time to last follow-up date, will be summarized in tables summarised using min, median and max, will be tabulated as number and percentage, place and individual characteristics.

5.2. Baseline characteristics and treatment group comparability

- Categorical variables will be described as number and percentage by randomized groups.
- Numerical variables will be presented as mean, median, standard deviation and range.
- The frequency of missing data will be presented in a separate column for all the variables.
- No statistical tests (p-value) will be estimated in the baseline table.

5.3. Primary efficacy analyses

5.3.1. Primary analysis

The differences in primary outcomes (SCORE risk and Framingham Risk Score) at one year between the treatment groups will be measured and analysed using t-tests. As the group sizes are large (more than thousand), it is valid to assume normal distribution of the error around the mean value estimates in both the intervention and the control group. Complementary to the absolute difference in the Score, Cohen´s effect size will be derived for the difference in mean outcome values divided by a pooled standard deviation of the Score between the 2 groups. A two-sided p-value <0.05 will be regarded as statistically significant.

5.3.2. Sensitivity analyses

Adjusted analysis will be conducted using linear regression with covariates. This is because many of the covariates are related to the prognosis of outcome (including sex and age) are related to the prognosis of outcomes, and in such situations, adjustments can yield coefficients slightly further from 0. We will use the continuous form of the primary outcomes (SCORE risk and Framingham Risk Score) as outcome in the regression analysis. We will assess the effects of the intervention independently for each of the outcome measure.
We will also conduct unadjusted linear regression as sensitivity analysis. Comparing the unadjusted and adjusted results provides assurance of the robustness of the results, especially when one of the results achieves only borderline significance.

We will conduct sub-groups analyses to compare the effects of the intervention among different population groups. More details about the sub-group analyses are presented in Section 5.4.1.

5.3.3. Effects in groups defined by their vascular ultrasound result

- Pairwise t-test will be used to assess the intervention effect in groups stratified by the vascular ultrasound result at baseline. The vascular ultrasound measurements includes information of the plaque presence and the age and sex standardized vascular age of the subject.
  For this test, the vascular age groups will be created according to the age standardized quartiles: green (young vascular compared to actual age), yellow (intermediate towards young), orange (intermediate towards red), red (old vascular compared to actual age). The t-test analysis will compare red to red, orange to orange, yellow to yellow, green to green of the intervention and control group, respectively. The t-test will be adjusted for multiple testing using the Bonferroni method.

5.3.4. Checking of assumptions

Only assumption is normal distribution which can be assumed for mean values estimates if groups are larger than 30. Since our groups is much larger than 30 we see not need to assess this assumption. We will calculate the t-test using the group specific standard deviations, to relax the assumption of equal variances.

5.4. Secondary outcome analyses

5.4.1. Sub-group analyses

In the analysis, we will compare the primary and secondary outcome variables' mean value between the intervention and the control in pre-specified sub-groups.

The sub-groups are:
- Abdominal obesity based on waist circumference measurement to compare the non-obese and obese groups, using the cut-off of waist circumference ≥102 cm for men and ≥88 cm for women according to the WHO definition.
- General obesity based on the body mass index measurement to compare the underweight, normal weight, overweight and obese groups, using the cut-off points of 18.5, 25, and 30, respectively.
- Physical activity level to compare respondents with low and high-level of physical activity
- Sex to compare men and women
- Age group to compare respondents at age 40, 50 and 60 years old
- Education level to compare respondents with low, medium and high education level as defined earlier.

- Since participants are recruited into the study during the three years from mid-2013 to mid-2016, any change in physicians' prescribing behaviour over the course of the study will be evaluated by comparing prescriptions issued for lipid-lowering and anti-hypertensive medications during the first year after the baseline ultrasound examination as well as primary outcome among participants recruited during the first, second and third years

5.5. Data handling and analytical steps

The data is entered using Microsoft SQL and is stored in common database file formats including SPSS and Stata. We will follow the following steps in handling the data to ensure its validity and make an analytical dataset for the analysis of this paper.

Step 1: We will conduct **quality check** on the baseline and 1-year follow-up data. Information from baseline and 1-year follow-up will be merged using the unique study number allocated for each individual (the VIPVIZAID). Individuals who did not participate in the 1-year follow-up will be noted and reasons for not participating will be identified and listed in the dataset. The completeness of all variables will be checked through, and if needed, the research nurse will double check any missing values in the dataset against the individual's responses in the paper questionnaire.

Step 2: We will check the dataset for **outliers and illogical values**. For categorical variables, responses outside the possible response categories will be coded as missing. For continuous variables, values outside the plausible ranges will be coded as missing also.

For biological markers, we will use the following cut-off points to define outliers. Any values outside these ranges will be coded as missing.

Variables	Plausible values
Fasting blood glucose (mmol/L)	2.0 – 25.0
Serum cholesterol (mmol/L)	2.0 – 18.0
LDL cholesterol (mmol/L)	1.2 – 15.0
HDL cholesterol (mmol/L)	0.4 – 6.0
Serum triglycerides (mmol/L)	0.4 – 20.0
Systolic blood pressure (mmHg)	80 – 240
Diastolic blood pressue (mmHg)	55* – 150
	*unless if systolic blood pressure below 90
Pulse pressure (mmHg)	15-100
Height (cm)	110 – 210
Change in height from baseline to 1-yr exam (cm)	>4
Weight (kg)	40 – 200
Waist (cm)	50 - 170
Change waist from baseline to 1-yr exam > 5cm	Change weight >15 kg
Change weight from baseline to 1-yr exam >5 kg	Change waist >15 cm

Step 3: After all the data have been cleaned, we will conduct **imputation of missing data** (see section 5.6). To the best possible, we will impute the original variables, not the derived variables. For example, instead of imputing the variable body mass index, we will impute the variable weight and height which is used to calculate body mass index. See the next section for more detailed descriptions on missing data.

Step 4: After the data have been imputed, we will generate **derived variables** based on the imputed data. For example, body mass index will be calculated from weight and height in the imputed data.

5.6. Imputation of Missing Data

Assessment of missing data will be conducted using the missing data modules in the Stata Statistical Programme. The command *misschk*, *misstable* will be used to assess the patterns of missing data among all the variables included in the analysis. To ensure reproducibility of data analysis conducted in VIPVIZA, we plan to create one imputed dataset based on assessment of all variables in the dataset and subsequently use this imputed dataset for analysis of future papers. Information about number of study participants with missing data for each variable will be presented in the description table showing the baseline characteristics of the study participants in the intervention and control groups.

We will conduct multiple imputation based on Rubin (1987) and Schafer (1997) methods using the *mi* estimate command sets in Stata. In brief, multiple imputation involves three steps: (i) <u>imputation step</u>: selection of imputation model to generate 10-20 imputed complete datasets to capture the uncertainty of the imputation model; (ii) <u>estimation step</u> (completed-data analysis): the planned analyses are conducted separately on each imputed dataset; and (3) <u>pooling step</u>: the results obtained from the series of completed-data analyses are combined into a single multiple-imputation result.

The command ***mi estimate*** estimates model parameters from multiply imputed data and adjusts coefficients and standard errors for the variability between imputations (***the estimation step***). It runs the specified estimation command on each of the M imputed datasets to obtain the M completed-data estimates of coefficients and their variance–covariance matrix of the estimators (VCEs). It then computes MI estimates of coefficients and standard errors by applying combination rules to the M completed-data estimates (***the pooling step***). Methods and formulas for computation details are described in the Stata's Multiple Imputations' guide, available at
https://www.stata.com/manuals13/mimiestimate.pdf

If data is **missing not at random**, a thorough assessment between study respondents with and without missing data on specific variables will be conducted based on the available information such as sociodemographic variables. The intention is to identify if this non-ignorable missing data might introduce any systematic bias that might influence the results of the estimation. We will employ selection models and/or pattern mixture models as multiple imputation strategies. Under the condition of MNAR, the joint density of VIPVIZA's participant responses is not the same for participants with full and partially observed data. We will perform pattern mixture models by modelling the observed data, then model the

missing data as a modification of the observed data model. We will explicitly model the missing data distribution by first identifying different patterns of missing data and then including parameters in the outcomes model that capture this effect (Paddock et al. 2006).

We will also conduct sensitivity analysis by comparing the results with complete case analysis vs. results with multiple imputation with different sets of imputation.

Reference for multiple imputations:
Stata Multiple-Imputation Reference Manual Release 13, StataCorp LP, Texas: https://www.stata.com/manuals13/mi.pdf).

Paddock SM, Edelen MO, Wenzel SL, Ebener PA, Mandell W. 2006. Pattern-Mixture Models for Addressing Nonignorable Nonresponse in Longitudinal Substance Abuse Treatment Studies. RAND Health.

5.7. Safety Analysis

The intervention in VIPVIZA is pictorial information about ultrasound results on the individual's actual atherosclerosis. This is considered to be a low-intensity intervention in comparison to interventions with pharmacological drugs or surgical procedures. The ultrasound examination cannot cause any harm, physical discomfort or risk. As with all screening targeting a healthy population, it is a dilemma that asymptomatic individuals may be informed of silent disease, in this case ongoing atherosclerotic process with increased risk of future CVD. This can be perceived more serious than just an increased level of risk markers, and result in anxiety.

In order to avoid unjustified concerns, all persons in the intervention group receive a telephone call with a research nurse and, if necessary, a doctor in charge, for in-depth and balanced information about ultrasound results. This conversation is held according to the methodology of motivational interviewing and also aims at increased awareness of the possibility of reducing the individual risk by means of own preventive measures. This is expected to alleviate anxiety and increase motivation to follow the recommendations for preventive treatment.

According to the study hypotheses, this is expected to benefit the individual due to risk reduction. A healthier lifestyle is also expected to benefit the individual through increased well-being and quality of life. Similarly, subjects without ongoing atherosclerotic disease will be able to avoid unjustified concerns and informed to continue a healthy lifestyle.

All individuals with severe carotid stenosis will be excluded from the study and are referred directly to the Stroke Centre for assessment and treatment. This may potentially be life saving for these people, and may, to some extent, balance the fact that no information is given until after 3 years to half the group even if small/moderate changes are detected.

5.8. Other planned analyses

- In **medium-term**, adding risk communication using visualisation of carotid ultrasound results is more effective in reducing **hospitalisation due to stroke, myocardial**

infarctions and revascularisations (at 5-year and 10-year) compared to routine CVD risk assessment and control within the primary health care setting. – Not for 1-year evaluation.

- In **long-term**, adding risk communication using visualisation of carotid ultrasound results is more effective in reducing **overall mortality and cause-specific mortality due to myocardial infarctions and stroke** (at 5-year and 10-year) compared to routine CVD risk assessment and control within the primary health care setting. – Not for 1-year evaluation.

6. Descriptions of Sample Size

Calculations based on data on conventional risk factors derived from VIP 2011, revealed that 3500 included study participants with a drop-out rate of 15 % during the study would be sufficient to assure a probability of 80% to detect a true difference between groups at a significance level of 5%. The limiting factor (demanding the largest group size to show a hypothesized effect) was CIMT.

Variabel	Significance level	Power	SD	Relevant change Population level	Sample size per group	Minimum detectable change if n=1500/group
SBP (mmHg)	0.05	0.8	16	2	1500	1.7
Serum Cholesterol (mmol/L)	0.05	0.8	0.5	0.5	1500	0.05
LDL (mmol/L)	0.05	0.8	0.5	0.5	1500	0.05
CIMT (mm)	0.05	0.8	0.2	0.02	1500	0.02
SCORE	0.05	0.8	1.40	0.5	1500	0.143
Framingham Risk Score	0.05	0.8	6.60	1	1500	0.7

7. Analysis Database Definitions

Variable	Description
age_0	Age in days at VIP baseline (*Rec)
age_1	Age in days at 1 year examination (*Rec)
alder_0	Age group (*Rec)
byear_0	Year of birth
in_kon	Gender (*Rec)
in_part0	Eligible participant and participation in main study (baseline) (*Rec)
in_part1	Eligible participant and participation in 1 year follow-up (*Rec)
in_randg	Randomization group (*Rec)
in_status	Participation status
educa_0	Education status (*Rec)
health_0	Perceived health during last year
g1d_0	Travel to and from work - Winter (*Rec)
g1d_1	Travel to and from work - Winter (*Rec)
g1km_0	How many kilometers do you have to travel to commute? (One way) (*Rec)
g1km_1	How many kilometers do you have to travel to commute? (One way) (*Rec)
g3a_0	What recreational activities do you participate in - Walks (*Rec)
g3a_1	What recreational activities do you participate in - Walks
g3b_0	What recreational activities do you participate in - Bicycling (*Rec)
g3b_1	What recreational activities do you participate in - Bicycling
g6_0	How often have you worked out or exercised in your training-clothes during the last three months (*Rec)
g6_1	How often have you worked out or exercised in your training-clothes during the last three months (*Rec)
g9_0	To what extent have you been physically active during leisure time during the past 12 months? (*Rec)
g9_1	To what extent have you been physically active during leisure time during the past 12 months? (*Rec)
g10_0	During an ordinary week, how much time do you spend on moderately strenuous activities (*Rec)
g10_1	During an ordinary week, how much time do you spend on moderately strenuous activities (*Rec)
h1antal_0	Number of cigarettes / day
h1antal_1	Number of cigarettes / day
h1a_0	Do you presently smoke? No, I never have smoked
h1a_1	Do you presently smoke? No, I never have smoked
h1b_0	Do you presently smoke? Yes, I smoke cigarettes
h1b_1	Do you presently smoke? Yes, I smoke cigarettes
h1c_0	Do you presently smoke? Yes, I smoke cigars
h1c_1	Do you presently smoke? Yes, I smoke cigars

h1d_0	Do you presently smoke? Yes, I smoke a pipe
h1d_1	Do you presently smoke? Yes, I smoke a pipe
h1e_0	Do you presently smoke? Yes, I smoke occasionally (Not daily)
h1e_1	Do you presently smoke? Yes, I smoke occasionally (Not daily)
h1f_0	Do you presently smoke? Not now, but I used to smoke daily
h1f_1	Do you presently smoke? Not now, but I used to smoke daily
h1g_0	Do you presently smoke? Not now, but I used to smoke occasionally
h1g_1	Do you presently smoke? Not now, but I used to smoke occasionally
h4_0	Have you ever used snuff? (*Rec)
h4_1	Have you ever used snuff? (*Rec)
j01_0	How often do you have a drink containing alcohol?
j01_1	How often do you have a drink containing alcohol?
j02_0	How many drinks containing alcohol do you have on a typical day when you are drinking?
j02_1	How many drinks containing alcohol do you have on a typical day when you are drinking?
j03_0	How often do you have six or more drinks on one occasion?
j03_1	How often do you have six or more drinks on one occasion?
j04_0	How often during the last year have you found that you were not able to stop drinking once you had started?
j04_1	How often during the last year have you found that you were not able to stop drinking once you had started?
j05_0	How often during the last year have you failed to do what was normally expected of you because of drinking?
j05_1	How often during the last year have you failed to do what was normally expected of you because of drinking?
j06_0	How often during the last year have you needed a first drink in the morning to get yourself going after a heavy drinking session
j06_1	How often during the last year have you needed a first drink in the morning to get yourself going after a heavy drinking session
j07_0	How often during the last year have you had a feeling of guilt or remorse after drinking?
j07_1	How often during the last year have you had a feeling of guilt or remorse after drinking?
j08_0	How often during the last year have you been unable to remember what happened the night before because of drinking?
j08_1	How often during the last year have you been unable to remember what happened the night before because of drinking?
j09_0	Have you or someone else been injured because of your drinking?
j09_1	Have you or someone else been injured because of your drinking?
j10_0	Has a relative, friend, doctor or other health care worker been concerned about your drinking or suggested to cut down?
j10_1	Has a relative, friend, doctor or other health care worker been concerned about your drinking or suggested to cut down?
langd_0	Body height cm (*Rec)
langd_1	Body height cm (*Rec)

vikt_0	Body weight kg (*Rec)
vikt_1	Body weight kg (*Rec)
midja_0	Waist circumference cm (*Rec)
midja_1	Waist circumference cm (*Rec)
sbt_0	Systolic blood pressure mmHg (*Rec)
sbt_1	Systolic blood pressure mmHg (*Rec)
dbt_0	Diastolic blood pressure mmHg (*Rec)
dbt_1	Diastolic blood pressure mmHg (*Rec)
skol_0	Serum cholesterol mmol/l (*Rec)
skol_1	Serum cholesterol mmol/l (*Rec)
stg_0	Serum triglycerides mmol/l (*Rec)
stg_1	Serum triglycerides mmol/l (*Rec)
ldl_0	LDL-cholesterol mmol/l (*Rec)
ldl_1	LDL-cholesterol mmol/l (*Rec)
hdl_0	HDL-cholesterol mmol/l (*Rec)
hdl_1	HDL-cholesterol mmol/l (*Rec)
blods0_0	Blood sugar fasting mmol/l (*Rec)
blods0_1	Blood sugar fasting mmol/l (*Rec)
c10_1_0	Medication in the last 2 weeks - Hypertension (*Rec)
c10_1_1	Medication in the last 2 weeks - Hypertension (*Rec)
c10_5_0	Medication in the last 2 weeks - Lipid lowering drug (*Rec)
c10_5_1	Medication in the last 2 weeks - Lipid lowering drug (*Rec)
c10_8_1	Medication in the last 2 weeks - Diabetes medication (*Rec)
c2_0	Did any of your parents or siblings suffer a heart attack/myocardial infarction or stroke before age 60 years?
c3_0	Does any of your parents or siblings suffer from diabetes?
c6_0	Do you suffer from diabetes?
c7a_0	If you have answered Yes on question C6, what is your treatment? Diet and exercise
c7b_0	If you have answered Yes on question C6, what is your treatment? Tablets
c7c_0	If you have answered Yes on question C6, what is your treatment? Insulin
c7d_0	If you have answered Yes on question C6, what is your treatment? None of the above
smoking_0	Smoking
smoking_1	Smoking 1 year (*Rec)
snus_0	Use of snus
phyact_0	Physical activity (*Rec)
phyact_1	Physical activity 1 year (*Rec)
fruitandveg_0	Fruit and vegetable consumption (*Rec)
lrmx_0	IMT CCA max mean value independent of side and angle
lrplack_0	Plack left/right combined
imt_color_0	IMT color code for patient information
usage_0	Age in days at ultrasound baseline

usdat_0	Ultrasound date (*Rec)
provdat_0	Sample date on optical questionnaire
provdat_1	Sample date on optical questionnaire (*Rec)
