# Supplementary material for: Prescription of Lipid-Lowering and Antihypertensive Drugs Following Pictorial Information About Subclinical Atherosclerosis: A Secondary Outcome of a Randomized Clinical Trial
Source: JAMA Netw Open. 2021 Aug 19;4(8):e2121683. doi: 10.1001/jamanetworkopen.2021.21683 (PMC8377571; doi:10.1001/jamanetworkopen.2021.21683)
Supplement: Supplement 2. — eAppendix. Supplementary Information on the Ultrasound Examinations eFigure 1. Pictorial Information as Presented to Study Participants and Their Primary Care Physicians (Translated From Swedish) eTable 1. Operational Definitions of Variables eFigure 2. The CONSORT Flow Diagram eTable 2. Number of Subjects and Percentages With a First Prescription for Any Lipid-Lowering Drug Among Participants Without a Prescription at Baseline (n = 2962) in the VIPVIZA Study eTable 3. Number of Subjects and Percentages With a First Prescription for Any Antihypertensive Drug Among Participants Without a Prescription at Baseline (n = 2237) in the VIPVIZA Study [file jamanetwopen-e2121683-s002.pdf]

Supplementary Online Content

Sjölander M, Carlberg B, Norberg M, Näslund U, Ng N. Prescription of lipid-lowering and antihypertensive drugs following pictorial information about subclinical atherosclerosis: a secondary outcome of a randomized clinical trial. *JAMA Netw Open*. 2021;4(8):e2121683. doi:10.1001/jamanetworkopen.2021.21683

eAppendix. Supplementary Information on the Ultrasound Examinations

eFigure 1. Pictorial Information as Presented to Study Participants and Their Primary Care Physicians (Translated From Swedish)

eTable 1. Operational Definitions of Variables

eFigure 2. The CONSORT Flow Diagram

eTable 2. Number of Subjects and Percentages With a First Prescription for Any Lipid-Lowering Drug Among Participants Without a Prescription at Baseline (n=2962) in the VIPVIZA Study

eTable 3. Number of Subjects and Percentages With a First Prescription for Any Antihypertensive Drug Among Participants Without a Prescription at Baseline (n=2237) in the VIPVIZA Study

This supplementary material has been provided by the authors to give readers additional information about their work.

© 2021 Sjölander M et al. *JAMA Network Open.*

eAppendix. Supplementary Information on the Ultrasound Examinations

The same clinical scanner was used for all ultrasound examinations: Panasonic CardioHealth® Station (Panasonic Healthcare Corporation of North America, Newark, NK, USA). The system is equipped with a vascular software for automatic real-time cIMT measurement.

Real-time automatic edge-detection measurement of cIMT was performed in the distal 1-cm of the far wall of CCA at two predefined angles for insonation at each side (240°, 210° and 150°, 120° for left and right carotid artery, respectively) based on Meijer arc.

A carotid plaque was defined according to Mannheim plaque consensus[1], and decision of plaque occurrence was taken at the time of examination.

1. Touboul PJ, Hennerici MG, Meairs S, Adams H, Amarenco P, Bornstein N, Csiba L, Desvarieux M, Ebrahim S, Hernandez Hernandez R, Jaff M, Kownator S, Naqvi T, Prati P, Rundek T, Sitzer M, Schminke U, Tardif JC, Taylor A, Vicaut E, Woo KS. Mannheim carotid intima-media thickness and plaque consensus (2004-2006-2011). An update on behalf of the advisory board of the 3rd, 4th and 5th watching the risk symposia, at the 13th, 15th and 20th European Stroke Conferences, Mannheim, Germany, 2004, Brussels, Belgium, 2006, and Hamburg, Germany, 2011. Cerebrovasc Dis. 2012;34(4):290-6. doi: 10.1159/000343145. Epub 2012 Nov 1.

© 2021 Sjölander M et al. *JAMA Network Open.*

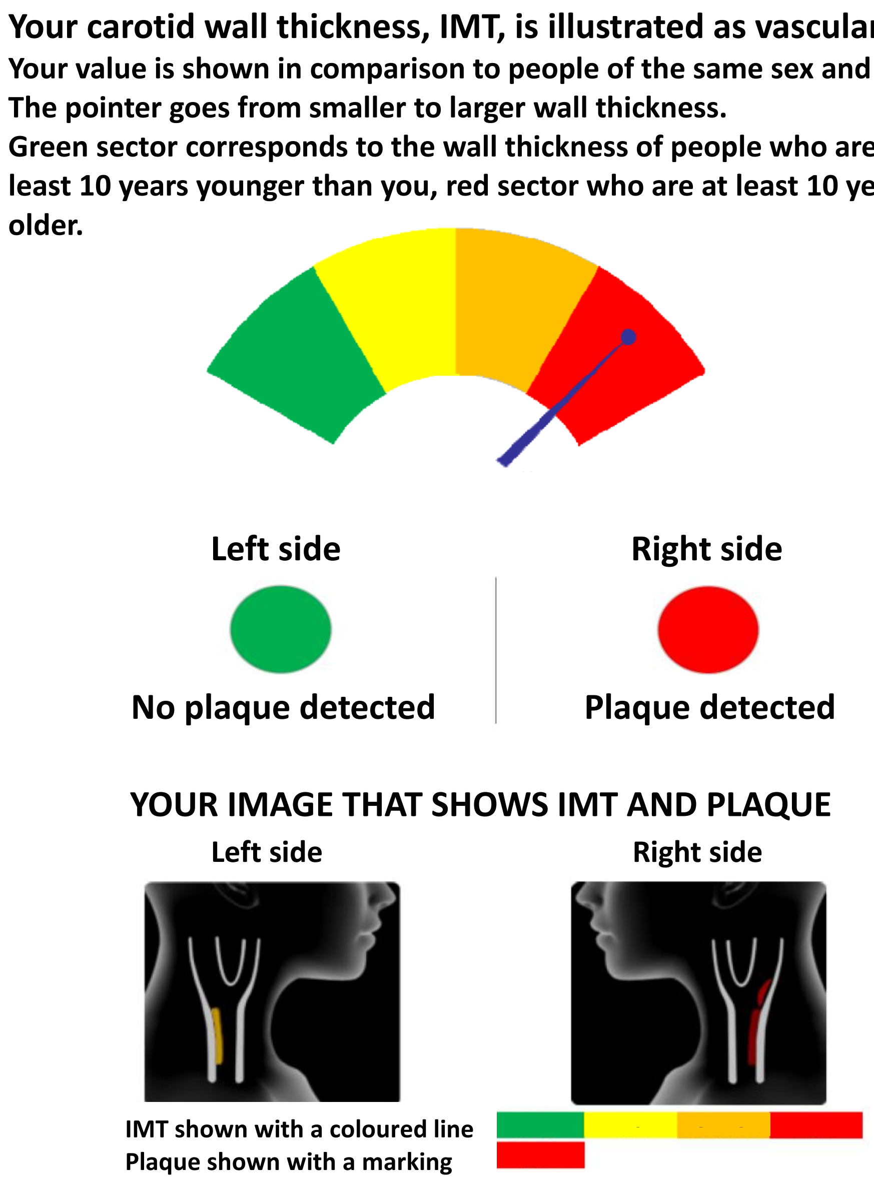

eFigure 1: Pictorial information as presented to study participants and their primary care physicians (translated from Swedish).

© 2021 Sjölander M et al. *JAMA Network Open.*

eTable 1. Operational definitions of variables.

Variables	Definitions
First prescription	Any prescription for any lipid-lowering (ATC codes C10) or antihypertensive drug (ATC codes C03A, C07, C08, C09) registered in the Region Västerbotten database, among participants without a prescription for a lipid-lowering drug within 465 days before ultrasound examination.
Education level	Self-reported level of education (Västerbotten Intervention Program (VIP) questionnaire)<br>- Low/middle: Basic education or high school<br>- High: university education
Previous myocardial infarction	Self-reported previous myocardial infarction (VIP questionnaire) Yes/No
Diabetes	Diabetes status from oral glucose tolerance test at health examination before ultrasound examination (VIP data) or self-reported diabetes (VIP questionnaire).<br>- Diabetes<br>- No diabetes (either normal or with Isolated Impaired fasting glucose (IFG) or with Isolated Impaired glucose tolerance (IGT) or with both IFG and IGT)
Systolic blood pressure	Systolic blood pressure measured at health examination before ultrasound examination (VIP data).
Diastolic blood pressure	Diastolic blood pressure measured at health examination before ultrasound examination (VIP data).
Serum cholesterol	Total serum cholesterol measured at health examination before ultrasound examination (VIP data).<br>- <5 mmol/l<br>- 5-6.5 mmol/l<br>- >6.5 mmol/l
LDL-cholesterol	Low-density lipoprotein measured at health examination before ultrasound examination (VIP data).
Prescription of antihypertensive drug at baseline	Any prescription for any antihypertensive drug (ATC codes C03A, C07, C08, C09) within 465 days before the ultrasound examination and registered in the county council database, Yes/No
Prescription of lipid-lowering drug at baseline	Any prescription for any lipid-lowering drug (ATC codes C10) within 465 days before the ultrasound examination and registered in the county council database, Yes/No
SCORE risk estimates	SCORE estimated absolute 10-year risk in %, at baseline (VIP data).
Year when participants were recruited to VIPVIZA	Study participants were enrolled over a three-year period.<br>- First year: April 2013 – June 2014<br>- Second year: July 2014 – June 2015<br>- Third year: July 2015 – June 2016
Vascular age	Ascertained by the measurement of carotid intima media thickness (cIMT) in relation to cIMT thickness for persons of same age and sex in the reference population (ARIC), and presented as vascular age indicated with a gauge from green to red.

© 2021 Sjölander M et al. *JAMA Network Open.*

	- Green: the intima media thickness corresponds to the average in persons at least 10 years younger than the chronological age<br>- Yellow: the intima media thickness corresponds to the average in persons of same chronological age. The closer the pointer is to Green, the more the thickness of the wall corresponds to a 5-10 years younger person.<br>- Orange: the intima media thickness corresponds to an average in persons of same chronological age. The closer the pointer is to Red, the more the thickness of the wall corresponds to a 5-10 years older person.<br>- Red: the intima media thickness corresponds to the average in persons at least 10 years older than the chronological age
Carotid plaque	Presence of any carotid plaque on one or both sides of the neck at the ultrasound examination. Yes/No

© 2021 Sjölander M et al. *JAMA Network Open.*

eFigure 2: The CONSORT flow diagram.

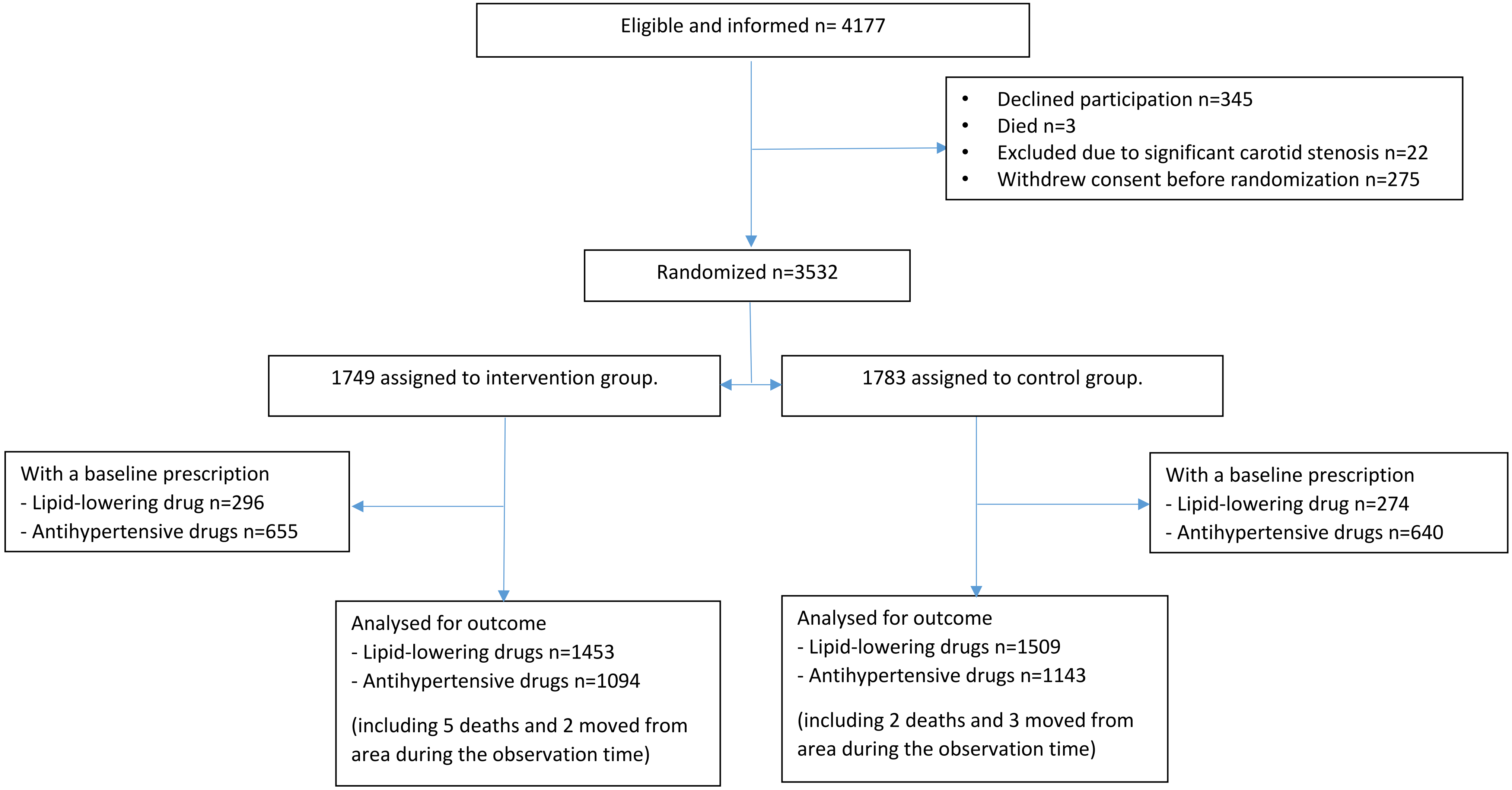

© 2021 Sjölander M et al. *JAMA Network Open.*

eTable 2: Number of subjects and percentages with a first prescription for any lipid-lowering drug among participants without a prescription at baseline (n=2962) in the VIPVIZA study. The results are presented for participants with or without carotid plaques and different vascular age (indicated by a gauge from Green through Yellow and Orange to Red with Green indicating a wall thickness of patients being at least 10 years younger than the actual age, and Red indicating at least 10 years older).

	**Control**	**Intervention**	**p-value**	**Control**	**Intervention**	**p-value**
	**Green/Yellow (n=531)**			**Orange/Red (n=1203)**		
No plaque (n=1734)	(n=273)	(n=258)		(n=601)	(n=602)	
First prescriptions for lipid-lowering drug within 465 days after the ultrasound (%)	4 (1.5)	13 (5.0)	0.019	32 (5.3)	61 (10.1)	0.002
	Green/Yellow (n=275)			Orange/Red (n=951)		
Plaque (n=1226)	(n=141)	(n=134)		(n=494)	(n=457)	
First prescriptions for lipid-lowering drug within 465 days after the ultrasound (%)	9 (6.4)	34 (25.4)	<0.001	31 (6.3)	136 (29.8)	<0.001

© 2021 Sjölander M et al. *JAMA Network Open.*

eTable 3: Number of subjects and percentages with a first prescription for any antihypertensive drug among participants without a prescription at baseline (n=2237) in the VIPVIZA study. The results are presented for participants with or without carotid plaques and different vascular age (indicated by a gauge from Green through Yellow and Orange to Red with Green indicating a wall thickness of patients being at least 10 years younger than the actual age, and Red indicating at least 10 years older).

	**Control**	**Intervention**	**p-value**	**Control**	**Intervention**	**p-value**
	**Green/Yellow (n=422)**			**Orange/Red (n=928)**		
No plaque (n=1350)	(n=217)	(n=205)		(n=462)	(n=466)	
First prescriptions for antihypertensive drug within 465 days after the ultrasound (%)	17 (7.8)	12 (5.9)	0.422	42 (9.1)	37 (7.9)	0.530
	Green/Yellow (n=193)			Orange/Red (n=692)		
Plaque (n=885)	(n=96)	(n=97)		(n=368)	(n=324)	
First prescriptions for antihypertensive drug within 465 days after the ultrasound (%)	12 (12.5)	11 (11.3)	0.804	49 (13.3)	57 (17.6)	0.119

© 2021 Sjölander M et al. *JAMA Network Open.*
