# Supplementary material for: Prescription of Lipid-Lowering and Antihypertensive Drugs Following Pictorial Information About Subclinical Atherosclerosis: A Secondary Outcome of a Randomized Clinical Trial
Source: JAMA Netw Open. 2021 Aug 19;4(8):e2121683. doi: 10.1001/jamanetworkopen.2021.21683 (PMC8377571; doi:10.1001/jamanetworkopen.2021.21683)
Supplement: Supplement 3. — Data Sharing Statement [file jamanetwopen-e2121683-s003.pdf]

Data Sharing Statement

Sjölander. Prescription of Lipid-Lowering and Antihypertensive Drugs Following Pictorial Information About Subclinical Atherosclerosis. *JAMA Netw Open*. Published August 19, 2021. doi:10.1001/jamanetworkopen.2021.21683

Data
Data available: No
